# Laminar Distribution of Phase-Amplitude Coupling of Spontaneous Current Sources and Sinks

**DOI:** 10.3389/fnins.2015.00454

**Published:** 2015-12-22

**Authors:** Roberto C. Sotero, Aleksandra Bortel, Shmuel Naaman, Victor M. Mocanu, Pascal Kropf, Martin Y. Villeneuve, Amir Shmuel

**Affiliations:** McConnell Brain Imaging Centre, Montreal Neurological Institute and Departments of Neurology, Neurosurgery, Physiology and Biomedical Engineering, McGill UniversityMontreal, QC, Canada

**Keywords:** spontaneous activity, resting state functional connectivity, local-field potentials (LFP), current-source density (CSD), cross-frequency coupling, phase-amplitude coupling, primary somatosensory cortex, cortical layers

## Abstract

Although resting-state functional connectivity is a commonly used neuroimaging paradigm, the underlying mechanisms remain unknown. Thalamo-cortical and cortico-cortical circuits generate oscillations at different frequencies during spontaneous activity. However, it remains unclear how the various rhythms interact and whether their interactions are lamina-specific. Here we investigated intra- and inter-laminar spontaneous phase-amplitude coupling (PAC). We recorded local-field potentials using laminar probes inserted in the forelimb representation of rat area S1. We then computed time-series of frequency-band- and lamina-specific current source density (CSD), and PACs of CSD for all possible pairs of the classical frequency bands in the range of 1–150 Hz. We observed both intra- and inter-laminar spontaneous PAC. Of 18 possible combinations, 12 showed PAC, with the highest measures of interaction obtained for the pairs of the theta/gamma and delta/gamma bands. Intra- and inter-laminar PACs involving layers 2/3–5a were higher than those involving layer 6. Current sinks (sources) in the delta band were associated with increased (decreased) amplitudes of high-frequency signals in the beta to fast gamma bands throughout layers 2/3–6. Spontaneous sinks (sources) of the theta and alpha bands in layers 2/3–4 were on average linked to dipoles completed by sources (sinks) in layer 6, associated with high (low) amplitudes of the beta to fast-gamma bands in the entire cortical column. Our findings show that during spontaneous activity, delta, theta, and alpha oscillations are associated with periodic excitability, which for the theta and alpha bands is lamina-dependent. They further emphasize the differences between the function of layer 6 and that of the superficial layers, and the role of layer 6 in controlling activity in those layers. Our study links theories on the involvement of PAC in resting-state functional connectivity with previous work that revealed lamina-specific anatomical thalamo-cortico-cortical connections.

## Introduction

Cross-frequency coupling describes statistical dependencies between the phases and/or amplitudes of different frequency bands of one or more signals. Three main modalities of cross-frequency coupling have been observed in local field potentials (LFP): phase-phase coupling (PPC), amplitude-amplitude coupling (AAC), and phase-amplitude coupling (PAC). It has been hypothesized that PAC of neurophysiological signals plays a mechanistic role in shaping neuronal oscillations and in the communication between cortical areas (Canolty and Knight, 2010).

PAC takes place when the phase of a low-frequency signal modulates the amplitude of a higher frequency one. Bragin et al. (1995) demonstrated a classic example of PAC in the CA1 region of the hippocampus, where the phase of the theta band modulates the power of the gamma band. More recent studies have shown that PAC is restricted neither to the hippocampus nor to theta-gamma (θγ) interactions. PAC has been detected in pairs of other frequency bands, including the delta, alpha, and beta rhythms in the cortex (Lakatos et al., 2005; Spaak et al., 2012; Wang et al., 2012; de Hemptinnea et al., 2013).

Our present study is motivated by two previously reported mechanistic theories that have been supported by experimental findings, and aims to connect between them. The first theory posits that neurophysiology-based resting-state functional connectivity (RSFC) is mediated by inter-areal synchronization of low-frequency (1–20 Hz; Lu et al., 2007; He et al., 2008; Wang et al., 2012) LFP. We note that infra-slow (< 0.5 Hz) fluctuations in LFP may also contribute to RSFC (Pan et al., 2013; see also our Discussion below). However, in the present study we focus on the contribution of low-frequency (1–20 Hz) LFP: we do not consider infra-slow (< 1 Hz) LFP. The observation that low-frequency LFP in the range of 1–20 HZ in remote areas are synchronized cannot account on its own for RSFC as measured with functional MRI (fMRI) (Biswal et al., 1995). The reason for this apparent inconsistency is that spontaneous fluctuations in fMRI signals correlate more consistently with locally measured fluctuations in the power of the gamma band of LFP (Shmuel and Leopold, 2008; Schölvinck et al., 2010; Pan et al., 2011) than with lower frequencies in the range of 1–20 Hz (Schölvinck et al., 2010; Pan et al., 2011). To resolve this apparent inconsistency, Wang et al. (2012) and Florin and Baillet (2015) suggested a model according to which RSFC is mediated by inter-areal synchronization of low-frequency LFP and by local PAC between the phases of these frequencies and the amplitude of the gamma band.

The second theory, based on anatomical studies (Felleman and Van Essen, 1991), posits that thalamo-cortical and cortico-cortical interactions occur in a lamina-specific manner. Moreover, the laminar structure of neo-cortical columns is thought to undertake a central mechanistic role in cortical information processing (Douglas and Martin, 2004).

Based on these two theories, we hypothesize that in the spontaneous activity state, PAC should show intra- and inter-lamina-specific interactions. However, with only a handful of studies describing PAC in coarsely delineated layers of the monkey auditory and visual cortices (Lakatos et al., 2005, 2007; Spaak et al., 2012), current knowledge on the laminar distribution of spontaneous PAC involving other frequency bands, other cortical areas, and other species remains sparse.

In the present study we recorded spontaneous LFP using laminar probes inserted perpendicularly to the cortical manifold in the forelimb representation of area S1 of the rat brain. We computed time series of lamina-specific current source density (CSD) and intra- and inter-laminar PAC of CSD for all possible pairs of the classical frequency bands in the range of 1–150 Hz. Our study links recent theories on involvement of PAC in resting-state functional connectivity with previously shown laminar-specific thalamo-cortico-cortical anatomical connections.

## Materials and methods

We conducted 2 sets of experiments. The primary set involved 12 cerebral hemispheres from 8 rats. A secondary set of experiments involving 4 hemispheres from 4 additional rats was conducted to create a prototypical pattern of current-source density. The surgical procedures were the same in each set of experiments.

### Animals and surgical procedures

All procedures were approved by the animal care committees of the Montreal Neurological Institute and McGill University and were carried out with great care according to the guidelines of the Canadian Council on Animal Care. Animals were housed under controlled environmental conditions, at 22 ± 2°C and with a 12 h light/12 h dark cycle (lights on from 7:00 a.m. to 7:00 p.m.). They received food and water *ad libitum*.

Data were obtained from adult male Sprague-Dawley rats weighing 250–300 g. Acute experiments were performed. The rats were initially anesthetized with xylazine (10 mg/kg i.p.; Bayer Inc., Canada) and ketamine (50 mg/kg i.p.; Wyeth, Canada). They were intubated and then placed in a stereotaxic frame. The anesthetic effect of ketamine and xylazine lasted approximately 30 min. During this time, we supplemented their effect with 0.6–1.5% isoflurane (Benson Medical Industries Inc., Canada). For the remaining duration of the surgical procedure, the rats were anesthetized with 1.5–2% isoflurane. We assessed anesthesia level by monitoring the heart beat and pain reflexes of the rats. During the surgery, we ventilated the rats with a mixture of medical air (80%) and oxygen (20%). EEG, heart rate, body temperature, and peripheral capillary oxygen saturation were monitored continuously. The scalp was incised to expose the skull. We fixed 2 stainless steel screws (2.4 mm length) to the skull, one above each hemisphere (AP – 5.00; L ± 2.50). The screws were used for EEG recordings, and one of them was also used as the reference and ground for the LFP recordings. The screws were positioned above the visual cortex, far away from area S1FL, with only a minor effect expected on our LFP recordings in S1FL.

We thinned the skull overlying the primary somatosensory cortex until it was soft and transparent. We performed a round-shaped craniotomy with a ~4 mm diameter, centered on the forelimb representation in area S1 (S1FL) according to a stereotaxic atlas (Paxinos and Watson, 2005). To insert the recording probe, we resected the dura while keeping the exposed cortex wet with Hank's Balanced Salt Solution (Invitrogen, Canada). At the end of the surgical procedure, we used silicone to construct a chamber around the opening and filled the chamber with Hank's Balanced Salt Solution. Following the surgery, just before the recordings, animals were injected subcutaneously with a single dose of buprenorphine (0.01–0.05 mg/kg; Schering-Plough, UK). An initial bolus dose of medetomidine (0.05 mg/kg; Pfizer Inc., Canada) was administered subcutaneously, then it was continuously administered subcutaneously (0.1 mg/kg/h) throughout the recordings. Isoflurane administration was stopped following the injection of buprenorphine and medetomidine.

Throughout the recordings, the intubated rats were ventilated (55–60 cycles/min; MRI1 ventilator, CWE, Ardmore, PA) with medical air, supplemented by 0–10% oxygen. The level of oxygen was determined according to pulse oximetry, measured using a lingual clip sensor (Nonin Medical, Plymouth, MN): peripheral capillary oxygen saturation was maintained at 95% or higher. Body temperature was kept at 37°C by means of a heating pad and automatic feedback from a rectal temperature sensor (Harvard Apparatus, Holliston, MA). We monitored the EEG closely to ensure a proper level of anesthesia, as measured by balanced low (delta and theta) and higher frequencies. Heart rate was maintained in the range of 240–310 beats/min.

### Electrophysiological recordings

In the primary set of experiments, a linear probe with 32 contacts equally spaced at 100-μm intervals (NeuroNexus Technologies, Ann Arbor, MI) was targeted to area S1FL using a manually driven micro-manipulator (David Kopf instruments, Tujunga, CA). We used stereotaxic coordinates (Paxinos and Watson, 2005; AP 0.00 mm, ML ±4.50 mm, DV −2.5 mm) to target S1FL, and we confirmed the precise target for the probe placement by optical imaging the blood-oxygenation response to forepaw stimulation under illumination of wavelength of 605 nm (Imager 3001, Optical Imaging Inc., Rehovot, Israel). The probe was inserted approximately orthogonal to the local cortical surface, as assessed by monitoring the penetration angle from multiple points of view. To ensure coverage of the cortex by the contacts, we monitored the root-mean square (RMS) of the signals detected by the bottom-most contact. While we were driving the probe into the cortex, a decrease in the RMS marked the point at which the bottom-most recording contact reached the cortex. We then slowly lowered the probe 2.7 mm deeper, so that it spanned the entire thickness of the cortex. This left 2 or 3 contacts below (in white matter) and above (in Hank's Balanced Salt Solution) the cortex. Neurophysiological signals were sampled at 24414.1 Hz using a multi-channel recording system (RZ2; Tucker Davis Technologies, Alachua, FL) that enables reliable recordings of the multiband signals from 0.5 Hz to ~5 kHz.

### Experimental paradigm

**Figure 1:**
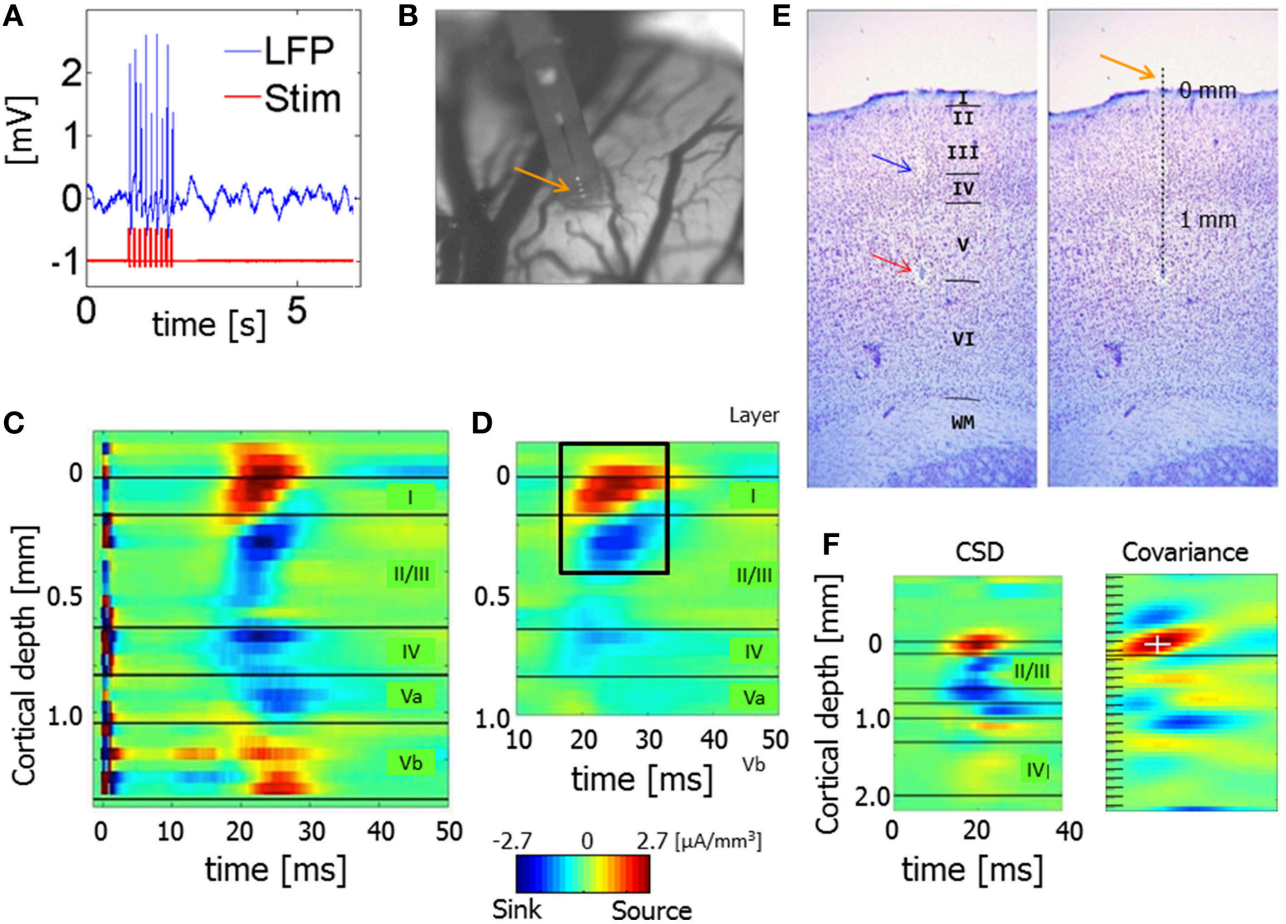
**Experimental paradigm and alignment of probes**. **(A)** LFP obtained from one channel positioned approximately 800 μm below the surface of the gray matter during a single stimulation trial. The red curve presents triggers used for forepaw stimulation. The evoked response appears shortly after each forepaw stimulus pulse. **(B)** Precise localization of laminar probes relative to the cortical surface: high-resolution image showing the part of a laminar probe close to the surface of cortex. Gray regions and dark curves around the probe are gray matter and pial blood vessels, respectively. Four contacts can be observed above the cortical surface, as indicated by the orange arrow. In this experiment and all other experiments conducted to create the prototypical CSD response presented in **(D)**, we used linear probes with 32 contacts densely spaced at 50 μm intervals covering layers 1–5. **(C)** The mean CSD response obtained from one run immediately after the image presented in **(B)** was acquired. The dark horizontal lines mark the approximate depth of the borders between the cortical layers, labeled on the right. Note that in this experiment we used a linear probe with 32 contacts densely spaced at 50-μm intervals covering layers 1–5. CSD values are in micro-Amperes per cubic millimeter. **(D)** The spatiotemporal pattern of mean CSD responses, used as a template for localizing CSD responses relative to the cortical surface. CSD responses from 12 runs in 4 animals were aligned relative to the cortical surface as determined by their corresponding images of contacts above the surface. The image represents the average CSD response computed following this alignment procedure. The prototype pattern used to align the results from the other experiments was extracted from within the dark rectangle. **(E)** A 50-μm-thick section of the brain cut from the recording site shown in **(B)** and stained with Cresyl violet. The image to the left shows a lesion induced through the bottom-most contact (red arrow), traces left by the probe (blue arrow), and delineation to the cortical layers. The image to the right presents the position of all 32 contacts spaced at 50-μm intervals, including the 4 observed above the cortical surface (orange arrow). **(F)** CSD responses averaged over forepaw stimulations in a single run. The horizontal and vertical axes represent the time from stimulation in milliseconds and the cortical depth in millimeters. The highest dark horizontal line marks the cortical surface based on the spatiotemporal covariance computed on the right. CSD values are in micro-Amperes per cubic millimeter. The image to the right presents spatiotemporal covariance as a function of time and space. The maximal covariance is marked with a white cross. The position of the cortical surface relative to the maximal covariance is marked with a horizontal dark line.

Data obtained from each of the 12 hemispheres from the 8 rats used for the primary set of experiments included runs with forepaw stimulation interleaved with stimulus-free runs lasting 10 min each during which spontaneous activity was recorded. Figure S1 presents 2-s segments of the broadband LFP obtained from each of the 12 hemispheres. In stimulation runs, electrical stimuli were delivered to the forepaw using ring electrodes positioned at the wrist (anode) and 1 cm more proximally (cathode), and a stimulator-isolator (A365, WPI, Sarasota, FL). Stimulation runs consisted of ten 12-s trials, with a 12-s interval between trials. In each trial we started by recording baseline activity for 1 s, then we applied a 1-s stimulus, then we recorded 10 s of baseline activity (Figure 1A). The 1-s stimulus consisted of a train of 8 monophasic electrical pulses (8 Hz, 1 ms each, 1.0–1.5 mA) delivered to the forepaw. The current was adjusted to provide stimulation just above the paw movement threshold. We used data from the stimulation runs to align CSD responses to the cortical surface, which enabled us to conduct lamina-specific averaging across data sets (see below).

### Analysis of neurophysiological responses using the standard CSD method

Neurophysiological data were processed using custom-written code in MATLAB. The recorded extracellular potentials were down-sampled to 24414.1/5 = 4882.8 Hz following low-pass filtering with a 10th-order finite impulse response (FIR) filter to prevent aliasing. LFP tend to be susceptible to volume conduction effects. To substantially reduce these effects, and to quantify PAC of the underlying causes of the LFP, we estimated PAC of current sources and sinks. For this, we used a second-derivative approximation for the CSD analysis (Mitzdorf, 1985). To obtain the spatiotemporal pattern of current sources and sinks, we applied the standard CSD estimation algorithm based on the second partial derivative of the laminar LFP along the cortical depth (Freeman and Nicholson, 1975; Mitzdorf, 1985). Assuming that the extracellular medium is resistive (ohmic) and uniform and that neuronal current sources and sinks extend over infinitely large planes with no variation in neuronal activity parallel to the cortical surface, the following 1-dimensional CSD equation holds (Mitzdorf, 1985):

(1) $${\tilde{I}}_{z}=-\lambda \frac{\partial^{2}\phi_{z}}{\partial z^{2}}$$

where *Z* is the axis of measurement, ϕ is the recorded LFP, *Ĩ*_*z*_ is the CSD distribution, and λ = 2.8*mS*∕*cm* (Goto et al., 2010). To calculate the second derivative of ϕ, we used the approximation proposed by Freeman and Nicholson (1975):

(2) $$\frac{\partial^{2}\phi }{\partial z^{2}}=\frac{\phi \left(z+2\Delta \right)-2\phi \left(z\right)+\phi \left(z-2\Delta \right)}{4\Delta^{2}}$$

where Δ is the distance between electrode contacts. CSD values at the top and bottom electrode contacts were obtained from Equation (2), under the assumption of constant or minimally decaying field potentials immediately above and below cortex as proposed by Vaknin et al. (1988). The most superficial recording site and the deepest recording site were used to provide the extra recording sites necessary to obtain a full description of the CSD distribution (Vaknin et al., 1988).

### Precise localization of laminar probes

To localize the contacts in each run relative to the cortical surface, we first created a prototypical high-resolution pattern of CSD response, computed using the standard CSD method and averaged over 12 stimulation runs in 4 animals in a secondary set of experiments. In this set of experiments, we conducted stimulation runs using the same paradigm as in the main set of experiments (Figure 1A). However, we used linear probes with 32 contacts more densely spaced, at 50-μm intervals, covering layers 1–5. After we observed a decrease in the RMS of the bottom-most recording contact, we lowered the probe 1.35 mm deeper, reaching the approximate position of the border between layers 5 and 6 and leaving ~4 contacts above the cortex (in the cerebrospinal fluid). Interleaved between the recordings, we obtained high-resolution images of the probe and the cortical surface around it using a Dalsa Pantera 1M60 camera (Imager 3001, Optical Imaging Inc., Rehovot, Israel) and a macro lens. Figure 1B presents such an image, with 4 contacts that can be observed above the cortical surface. Figure 1C presents the CSD response obtained from the run associated with the probe insertion presented in Figure 1B. We analyzed the CSD response separately for each run from each of the 4 rats. The spatiotemporal CSD patterns from each run and experiment were aligned in time according to the time of electrical stimulation. They were then aligned in space according to their depth relative to the surface of cortex. We determined the depth by counting the number of contacts imaged above the cortex in each run. Following the alignment in time and space, we computed the CSD response averaged from all 12 runs and the 4 rats in this set of experiments (i.e., a total of 48 runs), with precisely known depths relative to the cortical surface (Figure 1D). The CSD analysis revealed a noticeable short-latency current source in superficial contacts, with the adjacent current sink below. The averaged spatiotemporal CSD response obtained 17–32 ms after stimulation and from 150 μm above the cortical surface to 400 μm below it was used as the prototypical CSD response with known depth relative to the cortical surface (dark rectangle in Figure 1D). We used this prototypical response as a reference to precisely localize the linear probes used in the 8 rats in the primary set of experiments. Data obtained from each animal were aligned to the cortical surface by matching that animal-specific CSD profile to the CSD prototype shown in Figure 1D. Specifically, we computed spatiotemporal covariance between the CSD obtained in each specific run and the prototypical CSD pattern (Figure 1F).

We aligned the data from each of the 8 rats in the main set of experiments as follows. Given that adjacent contacts in the main experiment were separated by 100 μm (Figure 1F, CSD), we sampled the LFP data at intervals of 50 μm by averaging the time courses from each pair of adjacent channels, thus creating a new channel with interpolated data between each pair. CSD was obtained from the resampled data at intervals of 50 μm. Spatiotemporal covariance (Figure 1F, Covariance) was computed between each run-specific CSD response and the prototypical pattern presented in Figure 1D. The data sets we aligned included all 32 (63 interpolated) channels. We computed the covariance between the prototypical pattern and the run-specific response at a temporal sampling frequency of 1 kHz and depth interval of 50 μm. The maximum of this covariance image reflects the best fit between the prototype and the run-specific CSD response. We expect this maximum to co-localize with the uppermost contact of the prototypical CSD response (Figure 1D), namely 3 contacts above the surface. In all analyses, we observed a clear maximal covariance in one contact, enabling us to determine the depth of all contacts relative to the cortical surface, in each run.

Based on published data (Wise and Jones, 1977; Lamour et al., 1983; Dykes and Lamour, 1988; Jellema et al., 2004; Paxinos and Watson, 2005; Alonso-Nanclares et al., 2012) we estimated the average approximate depth range for each cortical layer in area S1FL (L1, 0–160 μm; L2/3, 160–640 μm; L4, 640–840 μm; L5a, 840–1040 μm; L5b, 1040–1370 μm; L6, 1370–2100 μm), which we projected onto the neurophysiological data. Note that these cortical depths are approximate, because of the variability introduced by the involved methodologies and also because cortical layers show variable thickness across subjects. Figure 1E presents a Nissl-stained section obtained from the region corresponding to the probe penetration shown in Figure 1B, with a lesion at the border between layers 5b and 6, approximately matching our prediction of the position of this lesion (bottom contact in Figure 1C) based on the average depth per layer we computed. The layer-specific CSD patterns from our experiments matched the lower-resolution patterns reported in other studies that combined CSD with histology of cortical layers (Jellema et al., 2004). All results presented in this paper that involve meta-analysis over runs and animals were obtained after we aligned the superficial contacts to the surface of the individual animal's brain. We aligned the data we obtained from each run of spontaneous activity to the surface of the cortex based on the alignment computed for the stimulation run just preceding it. We aligned data from different animals and hemispheres according to their position relative to the cortical surface and then averaged the results.

### Estimation of spontaneous CSD based on the standard and inverse CSD methods

The LFPs obtained during runs of spontaneous activity were down-sampled to 24414.1/5 = 4882.8 Hz following low-pass filtering with a 10th-order FIR filter. The 20-min spontaneous LFP obtained from each hemisphere (two runs of 10 min) were divided into 100 intervals of 12 s each. For each of these time segments, we computed the corresponding time series of the standard CSD using Equations (1) and (2).

The standard CSD method assumes that neuronal current sources and sinks extend over infinitely large planes with no variation of neuronal activity parallel to the cortical surface (Pettersen et al., 2006). However, experimental studies have shown that the effective diameter of the columnar activity can be small (~0.5 mm; Nicholson and Llinas, 1971) and therefore the standard CSD analysis might not be applicable. An additional problem is that in experiments with laminar electrodes, substances such as saline (with a high conductivity) or oil (with a very low conductivity) are commonly added to prevent drying of the brain. This will affect the measured potential generated by the underlying cortical current-source density. To handle these situations, Pettersen et al. (2006) proposed the inverse CSD (iCSD) method. There are 3 variants of the iCSD method: δ-source iCSD (which assumes infinitely thin current-source discs), step-iCSD (which assumes step-wise constant CSD between electrode contacts), and spline iCSD (which assumes a smoothly varying CSD). We found no appreciable differences between them, although the spline iCSD method was the slowest to compute. In this work we use the step-iCSD method (Pettersen et al., 2006):

(3) $$I=F^{-1}\Phi$$

Where vectors *I* and Φ represent the CSD and LFP, respectively. The matrix elements of *F* are given by:

(4) $$F_{ij}=\int_{z_{i}-\Delta /2}^{z_{i}+\Delta /2}\frac{1}{2\sigma }\left(\sqrt{{\left(z_{j}-z\prime \right)}^{2}+R^{2}}-|z_{j}-z\prime |\right)dz\prime$$

Where Δ is the distance between electrode contacts, z is the axis of measurement, and R is the radius of the cylinder used to model the spatial spread of the neuronal activity. Since we did not have data on R during spontaneous activity, we calculated cross-frequency coupling using CSD obtained from both the standard method and the iCSD method. We computed iCSD with 3 different diameters of sources and sinks, 0.5, 1, and 2 mm, assuming the conductivity of saline to be 1.654 S/m for contacts above the brain.

### Phase-amplitude coupling

Although, several methods have been developed to estimate phase-amplitude coupling (Canolty et al., 2006; Penny et al., 2008; Canolty and Knight, 2010; Tort et al., 2010), no gold standard has emerged. A problem common to all methods is the lack of a universal minimal interval duration to be used in all experimental settings that ensures unbiased detection of PAC. Thus, for each experiment and each method, it is necessary to perform a control analysis to assess the minimal duration that provides reliable detection (Tort et al., 2010). We selected segments of 12 s length after confirming that PAC could be measured reliably in this timeframe, in a reproducible and computationally efficient manner. Note that our statistical testing against surrogate data diminishes the probability that our findings reflect biased or false-positive detection.

For each 12-s interval, the CSD time series from each of the 21 contacts in the gray matter was band-pass filtered into the 7 frequency bands of interest: delta, δ, 1–4 Hz; theta, θ, 4–8 Hz; alpha, α, 8–12 Hz; beta, β, 12–30 Hz; low-gamma, γ_*l*_, 30–50 Hz; middle-gamma, γ_*m*_ 50–100 Hz; high-gamma, γ_*f*_, 100–150 Hz. To this end, we designed FIR filters using MATLAB's signal processing toolbox function *firls.m*. To remove any phase distortion, the filters were applied to the original time series in the forward and then the reverse direction using MATLAB's function *filtfilt.m*. To characterize the frequency content of spontaneous CSD as a function of cortical layer, we applied fast Fourier transform (FFT) and computed the magnitude spectra of the 0.5 mm iCSD signal (Figure S2).

The analytic representation *z*_*m*_(*t*) of each filtered signal *x*_*m*_(*t*) (where *m* = 1,.,7 stands for the index of the frequency band) was obtained using the Hilbert transform *H*(*x*_*m*_(*t*)):

(5) $$z_{m}\left(t\right)=x_{m}\left(t\right)+iH\left(x_{m}\left(t\right)\right)=a_{m}\left(t\right)e^{i\phi_{m}\left(t\right)}$$

where ϕ_*m*_(*t*), *a*_*m*_(*t*)are the instantaneous phases and amplitudes, respectively, for the frequency band *m*. Amplitudes were normalized by subtracting the temporal mean and dividing the result by the temporal standard deviation to create the set of normalized band-passed signals. Normalization was done to facilitate comparison between different frequency bands.

To calculate the coupling between the phase ϕ_*lm*_ of the frequency band *m* in contact *l* and the amplitude *a*_*jk*_ of the frequency band *k* in contact *j*, we used the modulation index introduced by Canolty et al. (2006), which is based on the complex variable:

(6) $$y_{jl,km}\left(t\right)=a_{jk}\left(t\right)e^{i\phi_{lm}\left(t\right)}$$

The absolute value of the mean vector is given by:

(7) $$M_{jl,km}=\left|\frac{1}{N}\sum_{t}y_{jl,km}\left(t\right)\right|$$

where *N* is the number of data points in the interval. A significance value is attached to ${\tilde{M}}_{jl,km}$ using a surrogate data approach (Canolty et al., 2006). We created surrogate data by randomly shuffling the 100 amplitude segments. For each segment, we then computed its surrogate modulation index by using the segment's own phase time series and an instance (one segment) from the shuffled amplitude time series.

Note that to preserve the correlation structure of the data, we did not shuffle the data points within a segment; rather, we shuffled complete 12-s segments. This procedure was repeated twice, to produce 200 surrogate values.

For each of the 12 data-sets, a Z-score was then constructed as:

(8) $$Z_{jl,km}=\frac{\text{average}\left(M_{jl,km}\right)-\mu }{\sigma }$$

where average(*M*_*jl, km*_) is the average across segments, and μ and σ^2^ are the mean and variance obtained from the surrogate data. The average Z-score, $\bar{Z}$, was computed by averaging the 12 individual Z-scores from the 12 hemispheres. $\bar{Z}$ was compared to 0 using the 2-tailed *t*-test, while considering multiple comparisons with false discovery rate (FDR) *q* < 0.01 (Benjamini and Hochberg, 1995). For each PAC combination, the input to the FDR algorithm was a vector of 21^*^21 *p*-values associated with all pairs of contacts.

To compare the strength of the PAC between combinations of frequency bands, we performed hypothesis testing for each pair of PAC combinations from the 12 combinations that were different (higher) than 0 (**Figure 4**). For each combination, we selected the 10% (44 out of 441) pairs of contacts that showed the highest PAC (**Figure 4**). For each of the 12 hemispheres and each of the 12 combinations, we computed the average PAC over the 10% contacts selected for the combination. This resulted in 12 averaged PAC values per combination, one per each of the 12 hemispheres. For comparing the strength of two combinations, we tested the hypothesis that their strength was equal by applying matlab's function ttest2 (two tailed *t*-test, assuming the variances of the two samples were unequal). The inputs to this procedure were 2 vectors of 12 PAC values each; the 2 vectors corresponded to the PAC values associated with the compared combinations. The resulting 66 (12^*^11/2) *p*-values were corrected for multiple comparisons by employing FDR with *q* = 0.05 (Benjamini and Hochberg, 1995).

### Similarity coefficient between two spatiotemporal patterns of activity

To compare patterns of spatiotemporal CSD, we used a similarity coefficient between two matrices **A** and **B** as follows (Sotero et al., 2010):

(9) $$S=1-\frac{||A-B||}{\left||A|\right|+\left||B|\right|}$$

where $||\text{A}||=\sqrt{tr\left({\text{A}}^{T}\text{A}\right)}$ and $||\text{B}||=\sqrt{tr\left({\text{B}}^{T}\text{B}\right)}$ are the Frobenius norm of **A** and **B**, respectively. Note that by the triangular inequality:

(10) ||A+B||≤||A||+||B||

*S* is a measure of normalized distance between matrices. It can vary between 0 and 1. The latter value is attained if and only if **A** and **B** are identical.

## Results

**Figure 2:**
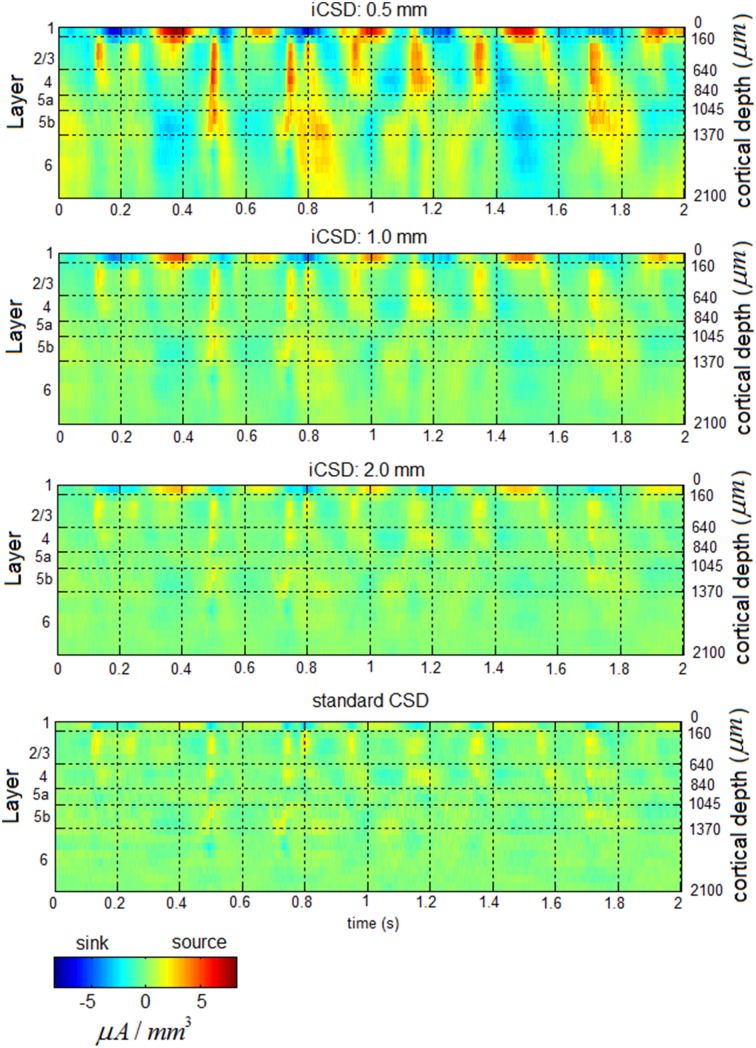
**Spontaneous current source density (CSD) from one hemisphere**. The 3 upper panels present the time courses obtained with the iCSD method for 3 diameters of sources and sinks: 0.5, 1, and 2 mm. The bottom panel presents the spontaneous CSD obtained using the standard method, which assumes infinite diameters of sources and sinks.

Figure 2 presents a 2-s time segment of CSD from one hemisphere. The data were obtained from 21 electrode contacts that spanned the cortical depth through all six layers (1, 2/3, 4, 5a, 5b, and 6). The time courses in the upper 3 panels were calculated for three different diameter sources (0.5, 1, 2 mm) using the iCSD method (Pettersen et al., 2006). The time course in the lower panel was calculated using the standard CSD method (Freeman and Nicholson, 1975), which assumes an infinite diameter of current sources. Although, the amplitudes of the sources and sinks were higher for smaller diameters, the spatiotemporal patterns were similar for the standard CSD and iCSD methods and across the assumed diameters for the sources and sinks. With increasing iCSD diameter (from 0.5 to 2 mm) the CSD spatiotemporal pattern became more similar to that of the standard CSD (consistent with the results of Pettersen et al., 2006), as reflected by similarity coefficients (Equation 9) values displayed in Table 1. Note that these values, which indicate the similarity between spatiotemporal CSD patterns, were computed using 12 second long segments from the entire 20 min data obtained from each hemisphere. To test whether these values could be obtained by chance, we split the standard CSD time-series to 12 s long segments, and randomly shuffled those segments. We then calculated the similarity coefficients between the non-shuffled 12 s long segments of iCSD with 0.5, 1, and 2 mm diameter and the shuffled standard CSD segments. This way, the surrogate data is constructed without affecting the temporal structure of the CSD matrices (Table 1). We concluded that the spatiotemporal patterns of spontaneous CSD were similar for the 2 methods and across the assumed diameters for the sources and sinks.

**Table 1:** **Similarity coefficients between iCSD and standard CSD for the 12 hemispheres**.

**iCSD 0.5 mm**		**iCSD 1 mm**		**iCSD 2 mm**	
**Similarity with standard CSD**	**Similarity with surrogate data**	**Similarity with standard CSD**	**Similarity with surrogate data**	**Similarity with standard CSD**	**Similarity with surrogate data**
0.597 ± 0.08	0.281 ± 0.08	0.668 ± 0.08	0.298 ± 0.08	0.795 ± 0.10	0.343 ± 0.10
0.565 ± 0.08	0.291 ± 0.08	0.676 ± 0.08	0.230 ± 0.08	0.769 ± 0.09	0.372 ± 0.10
0.580 ± 0.08	0.247 ± 0.08	0.674 ± 0.08	0.278 ± 0.08	0.802 ± 0.09	0.410 ± 0.10
0.545 ± 0.08	0.263 ± 0.08	0.666 ± 0.08	0.271 ± 0.08	0.777 ± 0.10	0.356 ± 0.10
0.543 ± 0.08	0.255 ± 0.08	0.699 ± 0.08	0.322 ± 0.08	0.850 ± 0.09	0.390 ± 0.10
0.583 ± 0.08	0.315 ± 0.08	0.671 ± 0.09	0.352 ± 0.08	0.824 ± 0.09	0.368 ± 0.10
0.508 ± 0.08	0.267 ± 0.08	0.682 ± 0.08	0.290 ± 0.08	0.763 ± 0.09	0.410 ± 0.09
0.513 ± 0.08	0.296 ± 0.08	0.700 ± 0.08	0.291 ± 0.08	0.799 ± 0.09	0.399 ± 0.10
0.517 ± 0.08	0.297 ± 0.08	0.654 ± 0.08	0.349 ± 0.08	0.794 ± 0.09	0.437 ± 0.10
0.539 ± 0.08	0.277 ± 0.08	0.733 ± 0.08	0.300 ± 0.08	0.803 ± 0.10	0.398 ± 0.09
0.583 ± 0.08	0.306 ± 0.08	0.690 ± 0.08	0.275 ± 0.08	0.782 ± 0.10	0.395 ± 0.10
0.580 ± 0.08	0.283 ± 0.08	0.653 ± 0.09	0.295 ± 0.08	0.796 ± 0.10	0.371 ± 0.10

Average similarity coefficients between iCSD and standard CSD for the 12 hemispheres. The similarity coefficients between iCSD with 3 different diameters of current sinks and sources (0.5, 1, and 2 mm) and the standard CSD were calculated using Equation (9) for each 12 s segment. To test whether these values could be obtained by chance, we calculated the similarity coefficients between 12 s long iCSD segments with the 0.5, 1, and 2 mm diameters and 12 s long standard CSD segments whose order was shuffled. This way, the surrogate data is constructed without affecting the temporal structure of the CSD matrices.

### Phase-amplitude coupling of current source density

**Figure 3:**
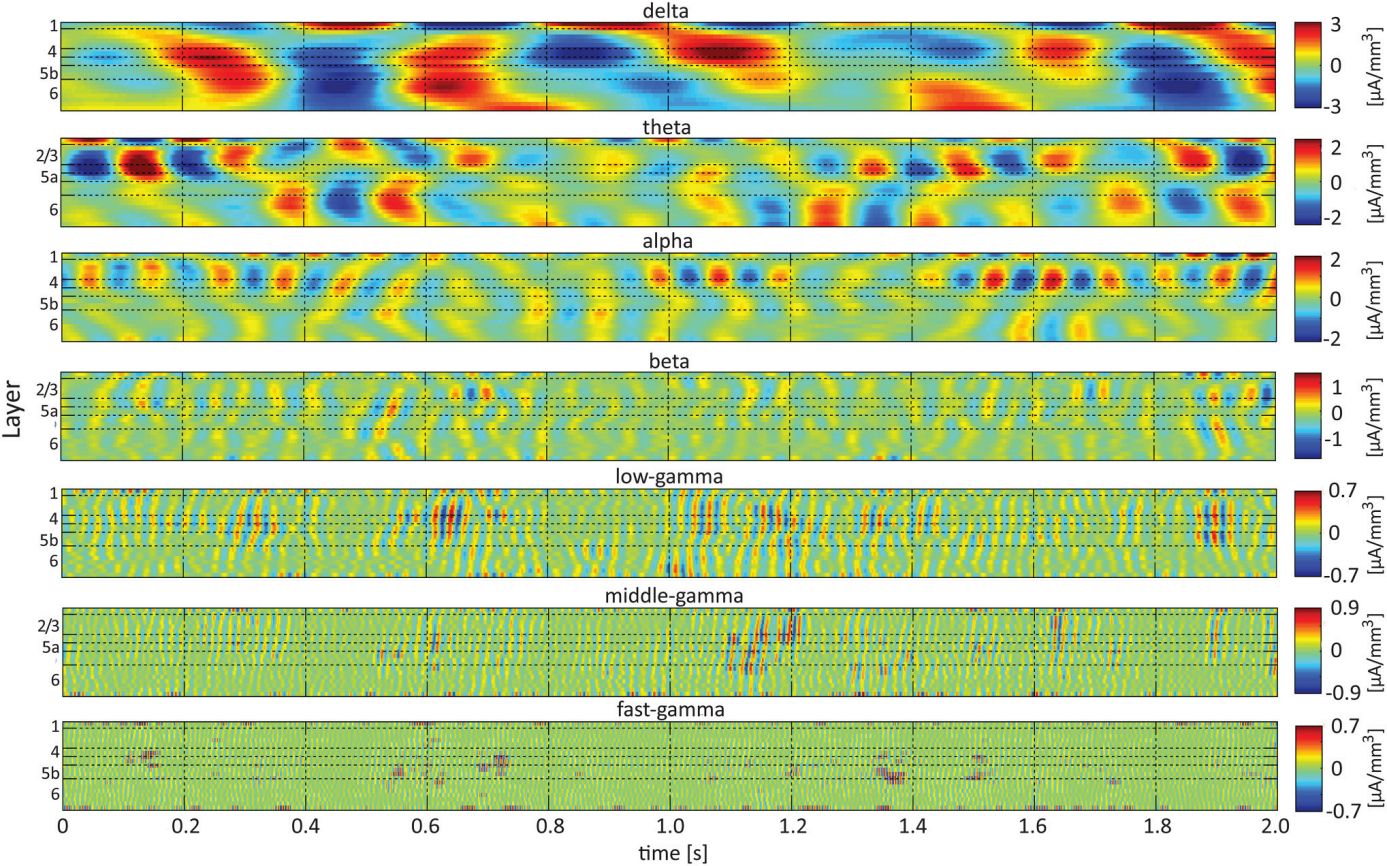
**Band-limited spontaneous current-source density**. The 7 panels show spontaneous current source density in the delta (**top**), theta, alpha, beta, low-gamma, middle-gamma, and high-gamma (**bottom**) bands. The color look-up table was scaled linearly according to the maximal absolute value, separately for each band.

Figure 3 presents band-limited spontaneous CSD obtained from a 2-s period. Alternating current sources (and separately, sinks) were approximately synchronized across layers 2/3–4 in the delta, theta, alpha, and beta bands. Current sources (and separately, sinks) in the theta and alpha bands in layers 2/3–4 and the middle-deep part of layer 6 appeared to be approximately 180° out of phase, with the reversals frequently occurring deeper than and adjacent to layer 5a. In addition, one can observe fluctuations in the amplitude of specific frequency bands, including the beta and gamma bands.

**Figure 4:**
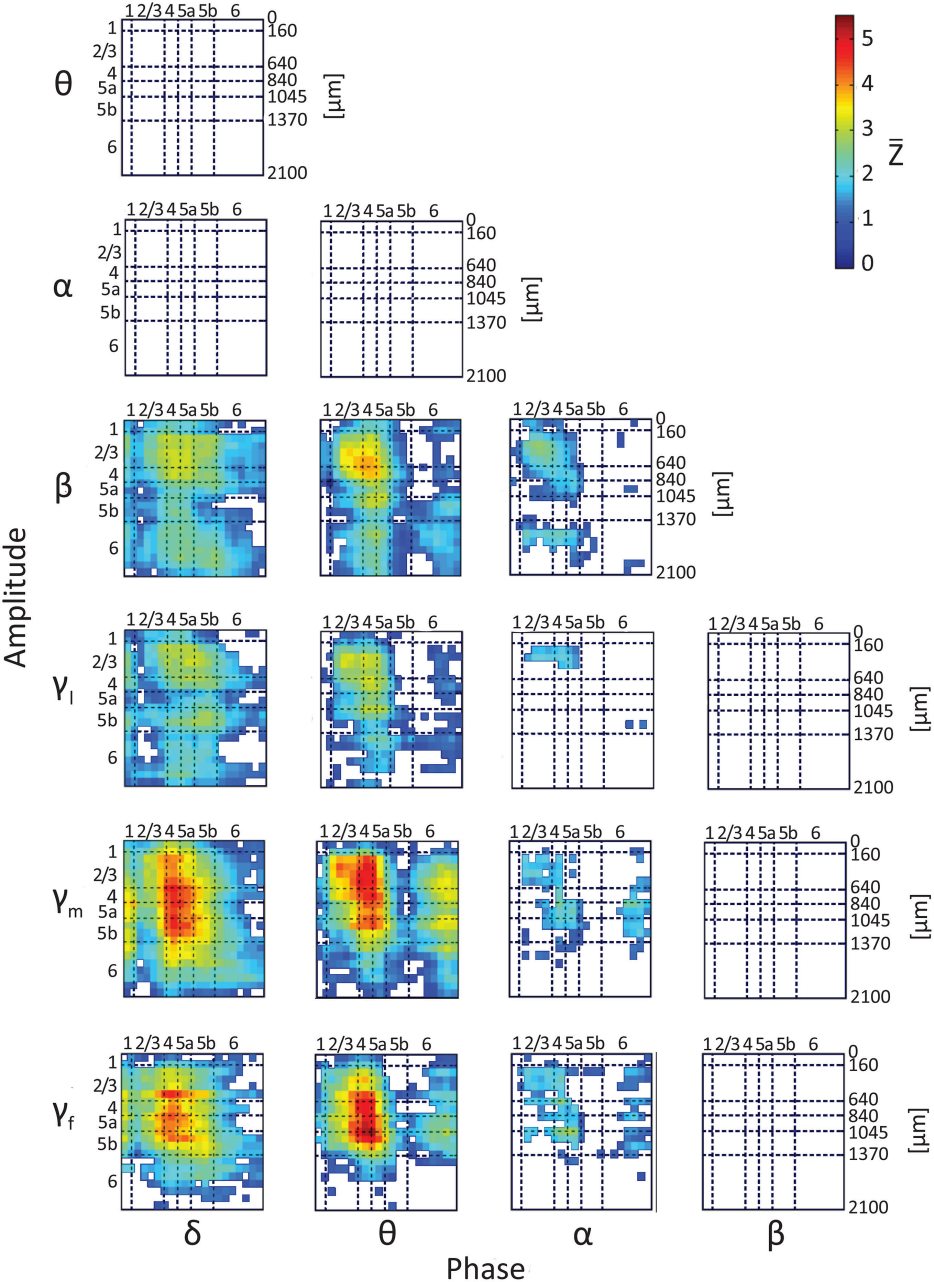
**Results of testing the null hypothesis that the average phase-amplitude coupling (PAC) is not different from 0**. The figure shows the average Z score, $\bar{Z}$, computed over 12 hemispheres (one Z-score per hemisphere) using iCSD and assumed 0.5-mm diameters of current sources and sinks. Seven frequency bands, delta (1–4 Hz), theta (4–8 Hz), alpha (8–12 Hz), beta (12–30 Hz), low gamma (30–50 Hz), middle gamma (50–100 Hz), and fast gamma (100–150 Hz), were considered. The PACs from the 18 different pairs of frequency bands we considered are presented in 18 different matrices. Each of the entries presents $\bar{Z}$, conditioned that it passed the statistical test, proving to be different from 0 (2-tailed *t*-test, while considering multiple comparisons with false discovery rate *q* < 0.01). White entries represent pairs of contacts that did not show statistically significant PAC.

To test whether the phases and amplitudes of band-limited spontaneous CSD interact, we computed lamina-specific PAC. Figure 4 presents intra- and inter-laminar PAC interactions averaged over 12 hemispheres $\left(\bar{Z}\right)$ for the CSD computed using the iCSD method and assuming a diameter of 0.5 mm for the current sources and sinks. The average PAC using iCSD and assumed diameters of 1.0 and 2.0 mm and standard CSD can be found in Figures S3–S5, respectively. For each pair of frequency bands, we calculated the modulation index between the phases of the slower rhythms (from delta to beta, see labels at the bottom of Figure 4) and the amplitudes of the faster rhythms (from theta to fast-gamma, see labels to the left of the panel in Figure 4). Each of these matrices has 21 rows and 21 columns and shows the average Z-score obtained from pairing a low-frequency rhythm from one of 21 electrode contacts with a high-frequency rhythm from one of these contacts. For each pair of frequency bands and each pair of contacts in each hemisphere, a modulation index was calculated for each of 100 12-s segments. The mean modulation index (computed by averaging over segments) was normalized to a Z-score by subtracting the mean and dividing by the standard deviation of modulation indices computed from the surrogate data. The 12 Z-scores obtained from the 12 hemispheres were then averaged to produce $\bar{Z}$. Each of the entries presents $\bar{Z}$, conditioned that it passed the statistical test, proving to be different from 0 (2-tailed paired *t*-test, while considering multiple comparisons with FDR, *q* < 0.01).

Of the 18 possible pairs of frequency bands, 12 showed statistically significant PAC (Figure 4). To compare the strength of the PAC among pairs of frequency bands, we performed hypothesis testing for each pair of PAC combinations selected from the 12 statistically significant combinations. Twenty-five of the 66 compared pairs of combinations were significantly different (Figure S6; 2-tailed *t*-test, while considering unequal variances associated with the two samples and correcting for multiple comparisons with FDR, *q* < 0.05). The 12 combinations ranked according to their mean PAC as follows:

$$\begin{array}{l} \theta \gamma_{f}>\theta \gamma_{m}>\delta \gamma_{m}>\delta \gamma_{f}>\theta \beta >\theta \gamma_{l}>\delta \beta >\delta \gamma_{l}>\alpha \gamma_{f}>\\ \alpha \beta >\alpha \gamma_{m}>\alpha \gamma_{l}. \end{array}$$

Although, the magnitudes and laminar distributions of these PACs quantitatively varied with the assumed source diameter for iCSD tangential to the cortical manifold, the description presented above remained qualitatively valid (Figures S3–S5). However, with increasing diameter of sources and sinks, the number of pairs of frequency bands showing statistically significant PAC decreased from 12 (for diameter 0.5 mm) to 11 (with standard CSD, assuming infinite diameter).

Note that there were no negative PAC interactions, indicating that for all pairs of frequency bands PAC in spontaneous activity was not lower than that of the corresponding surrogate data. The PAC interactions are listed below and are organized according to frequency band and layer, noting intra- and/or inter-laminar couplings.

#### Phase of delta

Phases of delta in layers 1–5b modulated amplitudes of the beta, low-gamma and middle-gamma bands throughout all cortical layers, and amplitudes of the fast-gamma band in layers 2/3–5b and to a lesser extent in layer 6. Phases of delta in layers 4–5a strongly modulated amplitudes of fast-gamma in the same layers and the amplitudes of the middle-gamma band throughout layers 2/3–5b. Phases of delta in layer 6 modulated amplitudes of the beta and gamma bands inconsistently through layers 2/3–6.

#### Phase of theta

Phases of theta in layers 2/3–5a modulated the amplitudes of the beta and gamma bands throughout layers 2/3–5b, and less consistently in layer 6. Phases of theta in layers 4–5a strongly modulated the amplitudes of middle- and fast-gamma in layers 2/3–5a. Phases of theta in layer 6 modulated the amplitudes of the middle gamma band throughout layers 2/3–6, and of the beta, low-gamma, and fast-gamma bands inconsistently throughout the same layers.

#### Phase of alpha

Phases of the alpha band in layers 2/3–5a modulated the amplitudes of the beta band in the same layers, amplitudes of the low-gamma band in layers 2/3, and the amplitudes of the middle- and fast-gamma bands inconsistently in layers 2/3–5a. Phases of the alpha band in layer 6 weakly modulated the amplitudes of the middle- and fast-gamma bands in layers 2/3–5b.

### Aligning amplitudes of high-frequency rhythms to the sinks and sources of the delta, theta, and alpha rhythms in the same contact

From the results presented in Figure 4, we concluded that the phases of the delta, theta, and alpha bands modulated the amplitudes of the higher frequency bands within and between laminae. This means that on average, the amplitudes of the higher rhythms depended on the phases of the lower rhythms. We next studied the nature of this dependence: is a sink in the lower rhythm associated with increases or decreases in the amplitude of the higher rhythm?

**Figure 5:**
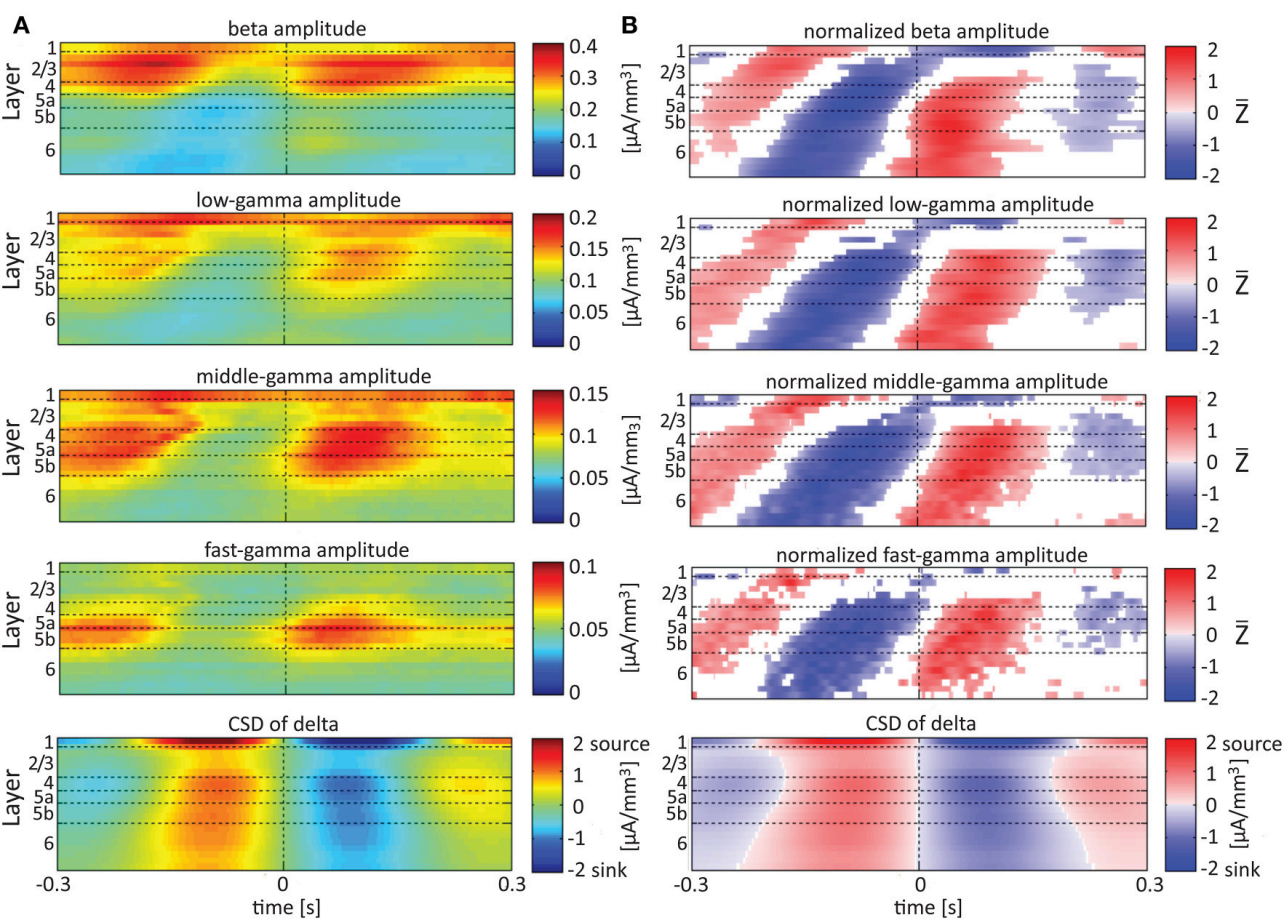
**Alignment of amplitudes with the CSD of delta oscillations**. In **(A)** the amplitudes of the higher rhythms (from beta to fast-gamma) in each contact were aligned with the CSD of the delta rhythm from the same contact. The reference signal, electrode contact-specific CSD in the delta band, is shown in the bottom panel. The CSD was calculated using the iCSD method with 0.5 mm diameter. In **(B)** the amplitudes presented in **(A)** were normalized contact-wise by subtracting the mean amplitude and dividing the result by the standard deviation of the amplitudes. White entries represent represent values that were not different than zero (*n* = 12 data-sets; two-tailed *t*-test corrected for FDR, *q* < 0.01).

**Figure 6:**
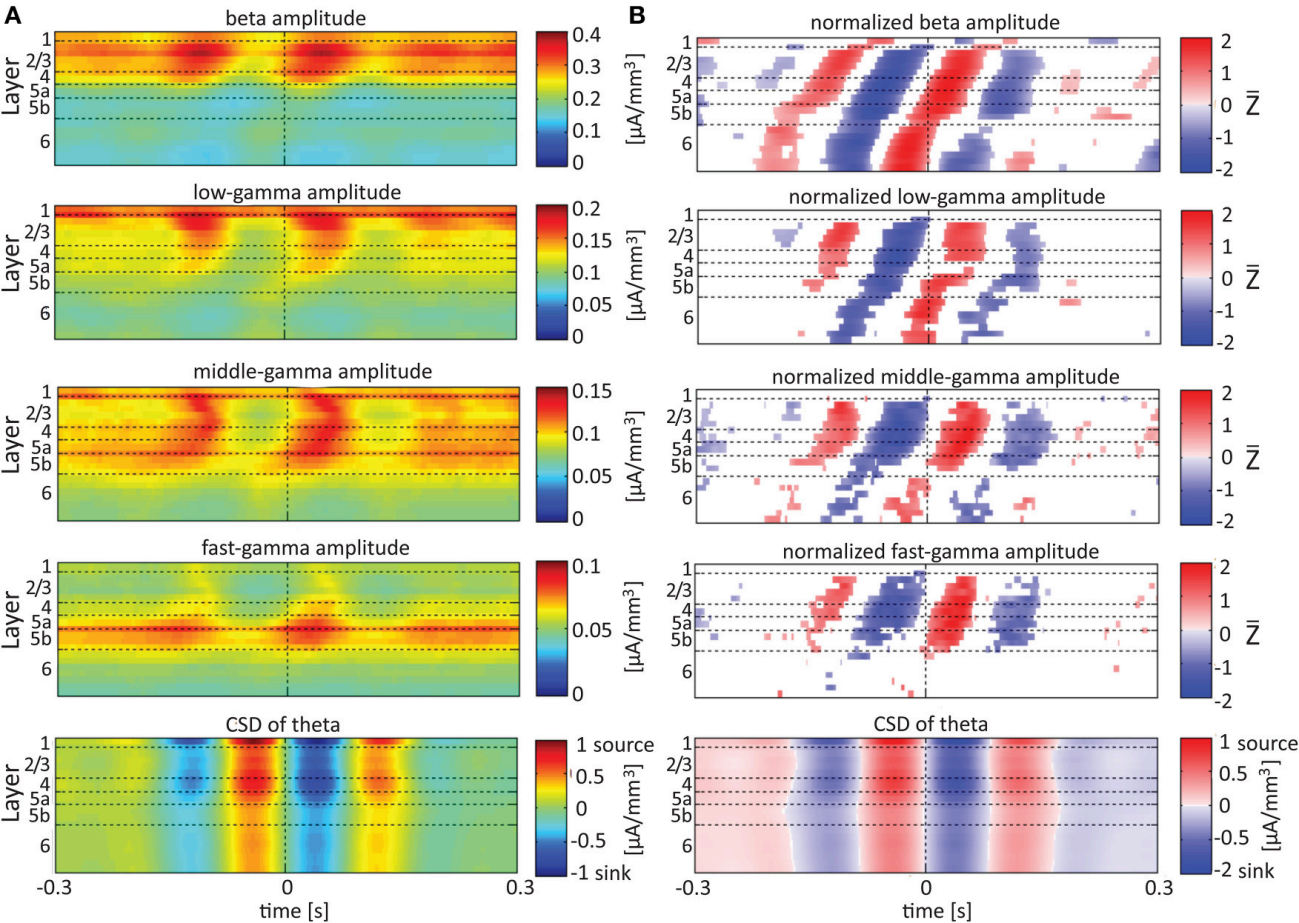
**Alignment of amplitudes with the CSD of the theta oscillations**. In **(A,B)** amplitudes and normalized amplitudes, respectively, of higher rhythms (from beta to fast-gamma) in each contact were aligned with the CSD of the theta rhythm from the same contact. The CSD was calculated using the iCSD method with 0.5 mm diameter. The normalization and format of presentation are identical to those used for Figure 5.

**Figure 7:**
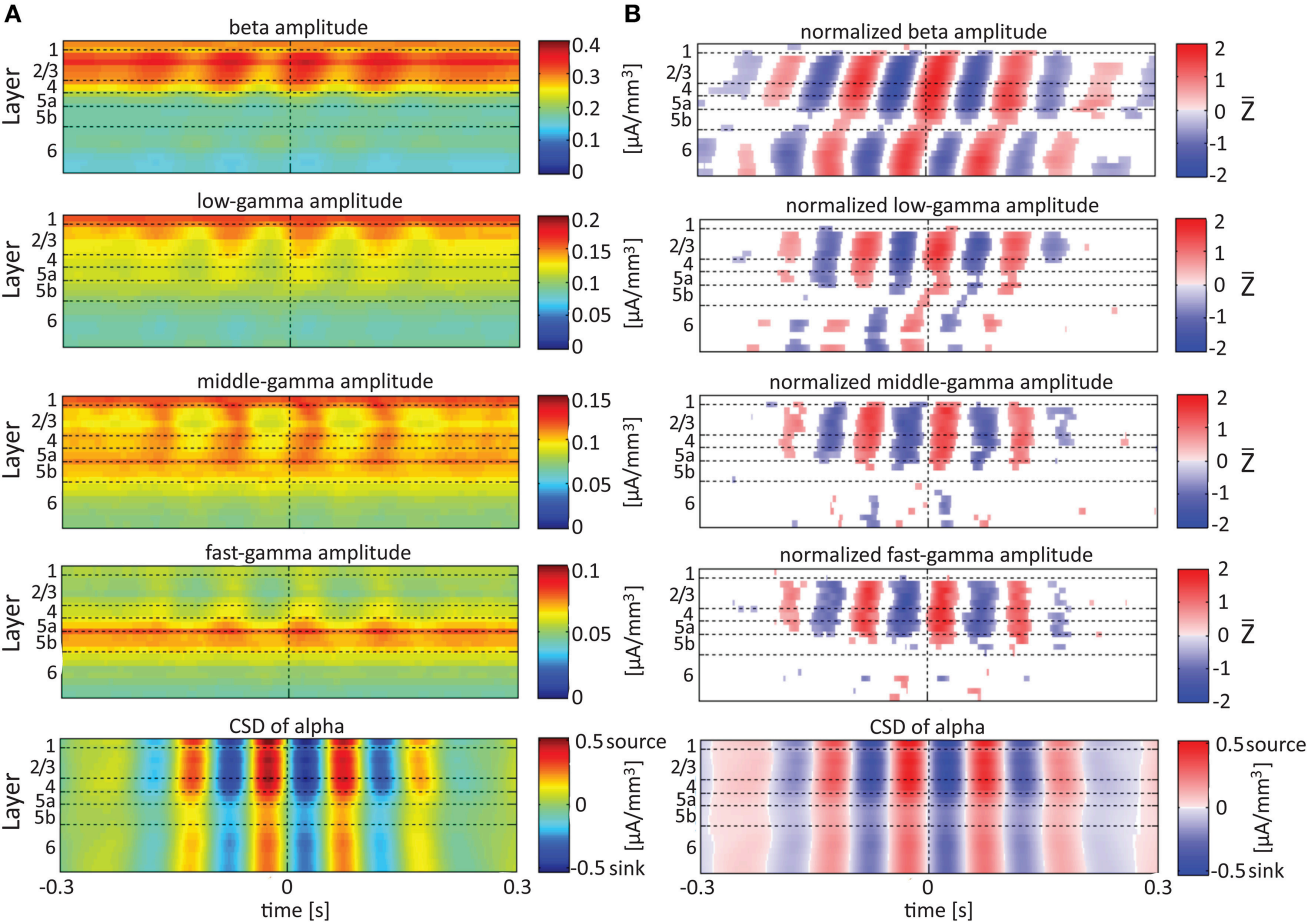
**Alignment of amplitudes with the CSD of the alpha oscillations**. In **(A,B)** the amplitudes and normalized amplitudes, respectively, of higher rhythms (from beta to fast-gamma) in each contact were aligned with the CSD of the alpha rhythm from the same contact. The CSD was calculated using the iCSD method with 0.5 mm diameter. The normalization and format of presentation are identical to those used for Figures 5, 6.

Single-trial CSD of spontaneous activity is noisy, and the lack of an external trigger makes ensemble averaging challenging. Using the signal from a contact as a reference overcomes this problem (see Bollimunta et al., 2008). Figures 5–**7** present the amplitudes of the faster rhythms (theta to fast-gamma) aligned with the sinks and sources of the delta, theta, and alpha bands, respectively. The designated alignment point, represented by 0 on the time axis, was the transition of the delta, theta, or alpha band CSD time series from source to sink in the same electrode contact. We averaged the corresponding CSD and amplitudes over time segments centered on such transitions. Figures 5–**7** present the averaged 600-ms segments centered on these transitions.

Referencing amplitudes to CSD sink and source in the delta rhythm (Figure 5) yielded increased and decreased amplitudes, respectively, in the beta and low-, middle-, and fast-gamma bands in layers 4–6 and to a lesser extent in layer 2/3. As can be seen in Figure 5B, there was no clear difference in the modulation of higher rhythms between layer 6 and superficial layers, except for near the transition from source to sink in the delta band. In contrast, when we referenced amplitudes to CSD in the theta and alpha bands (Figures 6, 7, respectively), the sinks (sources) in layers 2/3–5a were associated with high (low) amplitudes of the faster rhythm (beta to middle-gamma) in the same contact, while sinks (sources) in layer 6 were associated with low (high) amplitudes in the same contact.

### Aligning amplitudes of high-frequency rhythms to the sinks and sources of delta, theta, and alpha rhythms in specific layers

**Figure 8:**
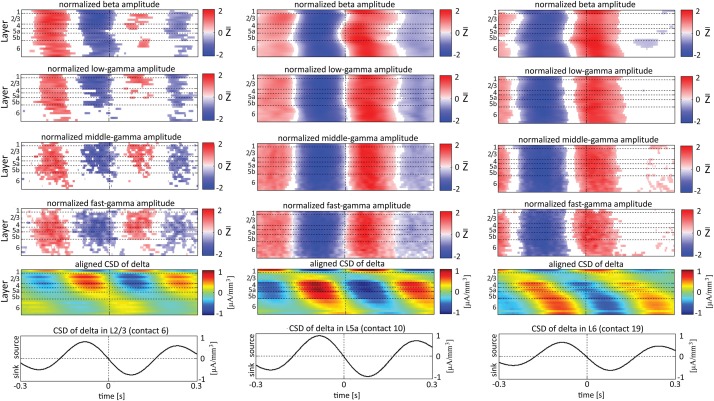
**Alignment of amplitudes with the CSD of the delta oscillations obtained from 3 specific contacts**. In the left-most columns, the amplitudes of higher rhythms (from beta to fast-gamma, top 4 panels) in each contact were aligned with the CSD of the delta rhythm from layer 2/3 (contact 6). The reference signal, the CSD in the delta band from contact 6, is shown at the bottom. The 2nd panel from the bottom presents the average of the CSD in the delta band aligned with the CSD of the same rhythm from contact 6. White entries in the panels that present amplitudes represent values that were not different than zero (*n* = 12 data-sets; two-tailed *t*-test corrected for FDR, *q* < 0.01). In the middle columns, the amplitudes of higher rhythms were aligned with the CSD of the delta rhythm from layer 5a (contact 10). In the right-most columns, the amplitudes of higher rhythms were aligned with the CSD of the delta rhythm from layer 6 (contact 19).

**Figure 9:**
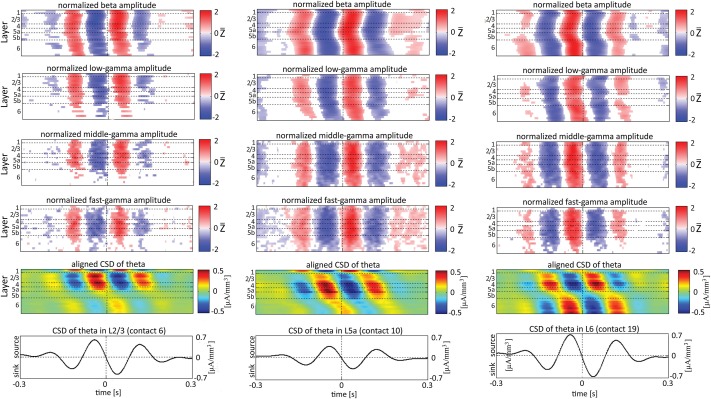
**Alignment of amplitudes with the CSD of theta oscillations obtained from 3 specific contacts**. The format of presentation is identical to that used for Figure 8.

**Figure 10:**
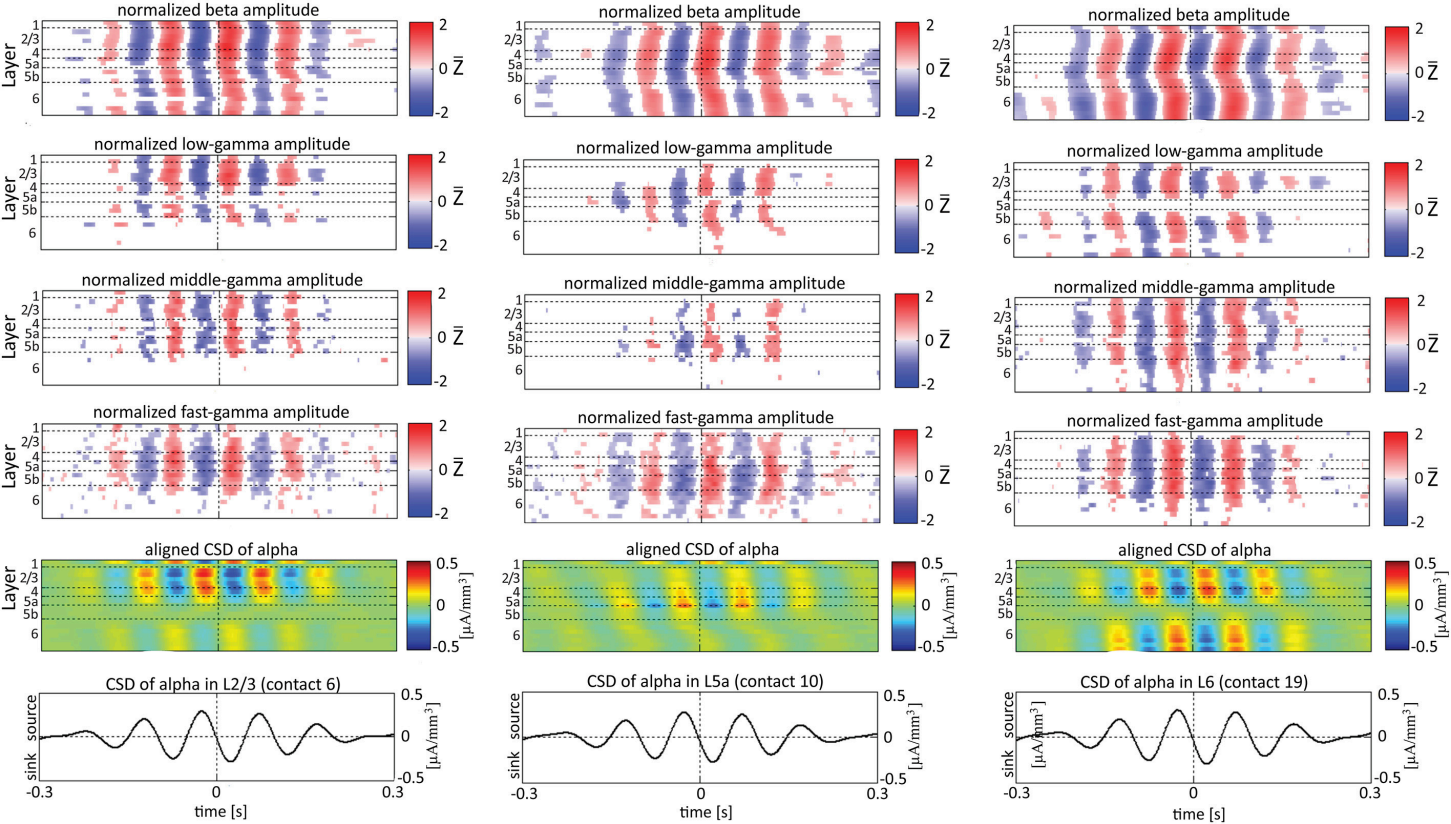
**Alignment of amplitudes with the CSD of alpha oscillations obtained from 3 specific contacts**. The format of presentation is identical to that used for Figures 8, 9.

Figures 5–7 present the amplitudes of the faster rhythms relative to the phases of the slower rhythms in the same individual recording contact. This analysis was pursued separately for each contact. However, the data shown in Figures 5–7 do not reflect the patterns of PAC over the entire column, from all contacts combined in a multivariate manner. To provide that information, we present the amplitudes of the faster rhythms (mean amplitude computed separately for each high-frequency band; see upper 4 rows in Figure 8) aligned with the sinks and sources of the delta, theta, and alpha rhythms in specific cortical layers in Figures 8–**10**, respectively (see the bottom row in each of Figures 8–**10**). To align the phase of the lower frequency band with the amplitude of the higher frequency band, we used as references specific contacts, which were approximately located in the middle of layers 3, 5a, and 6. These layers represent regions included in the superficial layers (layer 3), the deep layer (layer 6), and the approximate depth (layer 5a) just above the reversals we observed in band-limited CSD of the theta and alpha bands (Figure 3).

In addition to aligning amplitudes of the higher frequency bands to the transition between sources and sinks of the lower rhythm in specific cortical layers, Figure 8 also presents the lamina-specific CSD of the same lower rhythm over the entire column (see panels labeled “Aligned CSD of delta” in Figure 8), aligned to the same transition. Referencing amplitudes to CSD sink and source in the delta rhythm in layer 3 (Figure 8A) yielded increased and decreased amplitudes, respectively, in the beta and low-, middle-, and fast-gamma bands in layers 2/3–5b and to a lesser extent in layer 6. Note that the increased and decreased amplitudes that were aligned with sink and source in layer 3 were also associated with sinks and sources throughout layers 2/3–5a (see the panel labeled “Aligned CSD of delta” in Figure 8A). Referencing amplitudes to CSD sinks and sources in the delta rhythm in layers 5a and 6 (Figures 8B,C) yielded increased and decreased amplitudes, respectively, in the beta to fast-gamma bands in layers 2/3–6. Note that the increased and decreased amplitudes that were aligned with sinks and sources in layer 5a and layer 6 were also associated with sinks and sources throughout 4–6 (see the panels labeled “Aligned CSD of delta” in Figures 8B,C). Referencing amplitudes to CSD in the delta rhythm in layer 6 (Figure 8C) yielded increased and decreased amplitudes in the beta and gamma bands whose onsets were associated with transitions in layer 6 from source to sink and vice versa, respectively. We thus concluded that current sinks (and separately, sources) in the delta band were associated with increased (and decreased) cortical excitability throughout layers 2/3–6.

When referencing amplitudes to CSD in the theta band in layer 3 (Figure 9A), the sinks (sources) in layers 2/3 were associated with high (low) amplitude of the beta band in layers 2/3–6 and of the gamma band in layers 2/3–5b. Similarly, when referencing amplitudes to CSD in the theta band in layer 5a (Figure 9B), the sinks (sources) in layers 5a were associated with high (low) amplitude of the faster rhythms (beta to fast gamma) throughout layers 2/3–6. In contrast, sinks (sources) in layer 6 were associated with low (high) amplitudes throughout the same layers (Figure 9C). Referencing amplitudes to CSD in the alpha band (Figure 10) yielded qualitatively similar results to those observed for triggering on CSD in the theta band (Figure 9). Sinks (sources) of the alpha band in layers 2/3 and 5a (Figures 10A,B) were associated with high (low) amplitude of the beta band throughout layers 2/3–6 and of the gamma band in layers 2/3–5b. In contrast, sinks (sources) in layer 6 were associated with low (high) amplitudes of the faster rhythms throughout the same layers (Figure 9C). Note that sinks (sources) in layers 3 and 5a in the theta and alpha bands were on average associated with weak sources (sinks) in layer 6, creating a dipole centered on layer 5b (Figures 9, 10, panels labeled “Aligned CSD of theta and alpha”). Similarly, sinks (sources) in layer 6 were on average associated with sources (sinks) in layers 2/3–5a, creating a dipole centered on layer 5b. Therefore, the appearance of high or low amplitudes of the faster rhythms throughout the column were determined by whether the sinks in the theta and alpha bands were observed in superficial layers 2/3–5a or in layer 6, respectively.

Based on Figures 6, 7, 9, 10, we concluded that current sinks in the theta and alpha bands were associated with increases or decreases in cortical excitability depending on the cortical layer. We further concluded that sinks (sources) of the theta and alpha bands in layers 2/3–5a were on average part of a dipole completed by sources (sinks) in layer 6, associated with high (low) amplitudes of the faster rhythms from beta to fast-gamma in the entire cortical column.

## Discussion

Our findings can be summarized as follows. (1) There are significant intra- and inter-laminar PAC in a cortical column during spontaneous activity. (2) Statistically, significant PAC exists for all combinations of phases of the delta, theta and alpha bands and amplitudes of the higher rhythms, from beta to fast gamma; the highest PAC values were obtained for the combinations of phases of the theta and delta bands and the amplitudes of the middle- and fast-gamma bands. (3) Intra- and inter-laminar PAC involving layers 2/3, 4, and 5a show higher values than either intra- or inter-laminar interactions involving layer 6. (4) Current sinks and sources in the delta band are associated with increased and decreased cortical excitability, respectively, throughout layers 2/3–6. (5) On average, spontaneous current sinks (sources) in the theta and alpha bands in layers 2/3–5a are associated with high (low) amplitudes of faster rhythms from beta to fast-gamma in those same layers. Sinks (sources) in the theta and alpha bands in layer 6 are associated with low (high) amplitudes of faster rhythms. (6) Spontaneous sinks (sources) of the theta and alpha bands in layers 2/3–5a are on average linked to dipoles completed by sources (sinks) in layer 6, associated with high (low) amplitudes of faster rhythms from beta to fast-gamma in the entire cortical column.

### Lamina-specific PAC of spontaneous current source density

RSFC is associated with inter-areal synchronization of low-frequency LFP (1–20 Hz; He et al., 2008; Wang et al., 2012). Therefore, this synchronization is expected to play a role in mediating RSFC (Wang et al., 2012). However, spontaneous fluctuations in BOLD fMRI signals consistently correlate with fluctuations in the power of gamma-band LFP (Shmuel and Leopold, 2008; Schölvinck et al., 2010). In addition, fluctuations in the power of the gamma band demonstrate RSFC between the human auditory cortices of the two hemispheres (Nir et al., 2008) and separately between areas of the human somatomotor network (He et al., 2008). Therefore, Wang et al. (2012) and Florin and Baillet (2015) suggested that RSFC is mediated by inter-areal synchronization of low-frequency LFP associated with local PAC between the phases of these frequencies and the amplitudes of the gamma band.

Anatomical thalamo-cortical and cortico-cortical connections are expected to take an important role in mediating RSFC (Johnston et al., 2008; Zhang et al., 2008; Liu et al., 2012). The anatomical thalamo-cortical and cortico-cortical connections between sensory areas show lamina-specific origin of the projecting neurons (output from an area) and of the axonal terminals (input to an area; Felleman and Van Essen, 1991). These lamina-specific anatomical connection constrain the thalamo-cortico-cortical flow of neurophysiological activity in both the task- and resting-states. Therefore, if RSFC involves PAC, PAC can be expected to be lamina-specific. Consistent with this expectation and with PAC involvement in RSFC as proposed by Wang et al. (2012), Roux et al. (2013) and Florin and Baillet (2015), we demonstrate lamina-specific distribution of PAC between phases of low frequencies and amplitudes of the beta and gamma bands.

In addition to linking resting-state fluctuations in the BOLD signal to slow fluctuations in the power of gamma activity (Shmuel and Leopold, 2008; Schölvinck et al., 2010), a recent study demonstrated correlation between infra-slow (< 0.5 Hz) fluctuations in LFP and fluctuations in the BOLD signal (Pan et al., 2013). A follow-up study from the same group (Thompson et al., 2014) investigated the relationship between these infra-slow frequencies and higher frequencies under 2 anesthetics: isoflurane and dexmedetomidine. Isoflurane showed significant, consistent phase-amplitude coupling at nearly all pairs of frequencies. However, no consistent phase-amplitude coupling was observed in rats anesthetized with dexmedetomidine, which is similar to the medetomidine we used in the current study. In our current study we focus on PAC involving phases of low frequency (1–12 Hz) LFP, which are higher than the ultra-slow (< 0.5 Hz) LFP fluctuations studied by Pan et al. (2013) and Thompson et al. (2014). Therefore, our findings of robust lamina-specific PAC involving phases of slow (1–12 Hz) LFP and the amplitude of the beta and gamma bands are not at odds with the findings of Pan et al. (2013) and Thompson et al. (2014).

### Lamina-specific theta/alpha-phase dependent modulation of gamma-band activity

Our findings show that on average, spontaneous sinks in the theta and alpha bands in layers 2/3–5a are associated with an increase in the power of the beta and gamma bands in these layers. In contrast, sinks in the theta and alpha bands in layer 6 are associated with a decrease in the power of the beta and the gamma band (Figures 9, 10). Therefore, current sinks in the theta and alpha bands are associated with periodic excitability fluctuations that are lamina-dependent, with a transition in layer 5. Sinks (sources) of the theta and alpha bands in layers 2/3–5a are on average linked to dipoles completed by sources (sinks) in layer 6, associated with high (low) amplitude of faster rhythms from beta to fast-gamma in the entire cortical column (Figures 9, 10).

In rat area S1, layers 2/3, 4, and 5a mediate the feedforward flow of information via the lemniscal pathway (Herkenham, 1980; Lefort et al., 2009). In contrast, layer 6 mediates the feedback flow of information by receiving input from higher sensory areas (Felleman and Van Essen, 1991) and projecting to the thalamus (Bourassa et al., 1995). Here we demonstrated that CSD in the theta and alpha bands is in phase within layers 2/3–5a and separately in layer 6. On average, the CSD is in anti-phase between these 2 groups of layers (Figures 9, 10). In addition, the alpha/theta phase coupling with the amplitude of the beta and gamma bands in the feedforward layers is stronger than the PAC in layer 6. Thus, our findings show qualitatively different excitability patterns associated with theta and alpha PAC in the feedforward and feedback streams. Our results further support the dichotomy between the layers involved in the feedforward and feedback streams.

The highest level of PAC is observed for combinations involving phases of theta and delta in layer 4 (Figure 4 and Figures S3–S5). This indicates that the mechanisms may involve spontaneous ascending driving (Viaene et al., 2011) input from the thalamic VPM nucleus that projects a strong anatomical connection onto layer 4 (Herkenham, 1980, 1986; Lu and Lin, 1993; Bureau et al., 2006). However, Figures 9, 10 suggest that on average, PACs involving amplitudes in the beta band propagate from layer 5 to more superficial layers and to layer 6. This brings up the possibility of involvement of ascending connections from the POm, which shows relatively dense anatomical projections onto layer 5a (Herkenham, 1980, 1986; Lu and Lin, 1993).

Olsen et al. (2012) recently hypothesized that layer 6 acts as a gain controller in sensory areas. These authors showed that layer 6 in the primary visual cortex of the mouse plays a crucial role in controlling the gain of visually evoked activity in neurons of the upper layers without changing their tuning to orientation. This gain modulation results from the coordinated action of layer 6 intracortical projections to superficial layers and deep projections to the thalamus, with the intracortical circuit playing a substantial role. Our data show that layer 6 sinks in the theta and alpha bands are associated with sources in layers 2/3–4 and decreased amplitudes in the beta and gamma bands in layers 2/3–5a (Figures 9, 10). Thus, our findings suggest that the gain control by layer 6 involves activity in the theta and alpha bands and that it takes place also during spontaneous activity rather than just during evoked responses.

### Frequency bands definition and the extended (4–12 HZ) theta band

When comparing our results with previous studies, it should be taken into account that different animal studies use different frequency boundaries for their band definition. This is because the exact frequencies boundaries are unknown, and as a result frequency bands are usually selected based on human studies, intuition, or visual inspection of the data (Magri et al., 2012). Previous LFP recordings in rats suggest the presence of an “extended” theta band from 4 to 12 Hz (Jones and Wilson, 2005). Our findings support this notion, since we have demonstrated PAC between the phases of the theta and alpha rhythms and the amplitudes of the beta and gamma bands. Specifically, theta and alpha sinks in layers 2/3–5b are associated with higher beta and gamma power than their corresponding sources. Current sinks in the theta and alpha bands are associated with increased or decreased cortical excitability depending on the cortical layer (Figures 6, 7). Moreover, spontaneous sinks (sources) in both the theta and alpha bands in layers 2/3–5a are on average linked to dipoles completed by sources (sinks) in layer 6, associated with high (low) amplitude of faster rhythms from beta to fast-gamma in the entire cortical column (Figures 9, 10). The similar behavior of the theta and alpha bands during spontaneous activity consolidates their convergence to an extended band of frequencies.

Interestingly, analysis of LFP coherence patterns in monkeys suggests the existence of a similar extended band (6–16 Hz) that goes from high-theta to low-beta (Buffalo et al., 2011; Buschman et al., 2012; Spaak et al., 2012). The phase of this extended band may regulate information transmission in the cortex (Busch et al., 2009; Jensen et al., 2012).

### Hierarchy of CFC

Lakatos et al. (2005) reported significant δθ and θγ_*l*_ PAC in spontaneous CSD in the auditory cortex of the macaque. Their results revealed a hierarchical organization: the delta (1–4 Hz) phase modulated the amplitude of the theta band (4–10 Hz), and the theta phase modulated the amplitude of the gamma band (30–50 Hz). They proposed the existence of a hierarchical structure in the EEG, where the phase of each frequency band modulates the amplitude of a higher frequency band. They also proposed that this oscillatory hierarchy controls baseline excitability and that the hierarchical organization of ambient oscillatory activity allows the auditory cortex to structure its temporal activity pattern to optimize the processing of rhythmic inputs. Our findings suggest a somewhat different hierarchy of oscillatory activity with regard to these frequency bands. We did not observe PAC between the delta and theta bands. In rat area S1FL, PAC is statistically significant between the phases of the delta and theta bands and the amplitudes of the beta and gamma bands, but not between the phase of the delta band and the amplitude of the theta band (Figure 4). Our data support specific PAC interactions, but not a clear hierarchical PAC structure. The differences between Lakatos et al.'s findings and ours are consistent with their proposal that the hierarchical structure found in the auditory cortex may support processing of rhythmic auditory stimuli, which are less common in natural somatosensory stimuli to the forepaw.

### The spatial spread of current sources and sinks

Rather than analyzing the LFP, we chose to analyze the CSD, because this approach substantially reduces the effects of volume conduction. It therefore allows more direct interpretation of field potential oscillations in terms of the underlying synaptic activity in the local neuronal ensemble (Mitzdorf, 1985).

The spatial reach of the LFP is under debate, even for stimulation conditions (Katzner et al., 2009; Kajikawa and Schroeder, 2011). The lack of information concerning the size of current sources and sinks during spontaneous activity is even more significant. Without any definite information in this regard, we analyzed our data assuming 4 different diameters of current sources and sinks. We computed time series of lamina-specific CSD using the standard CSD method (Mitzdorf, 1985), which assumes an infinite diameter of current sources and sinks. To account for the finite spatial extent of current sources and sinks and the discontinuities in conductivity between the cortex and cerebrospinal fluid, we also computed the time series of inverse CSD (iCSD; Pettersen et al., 2006) for 3 different assumed diameters of the current sources and sinks: 0.5, 1, and 2 mm.

Concerning the noise associated with iCSD, from the perturbation theory we see that there is an upper bound to noise amplification associated with the inverse problem, which is given by:

(11) $$\left||\text{C}-{\text{C}}^{\prime }||/||\text{C}||<=\text{cond}(\text{F})||\text{V}-{\text{V}}^{\prime }||/||\text{V}|\right|$$

where cond(F) is the condition number of the forward matrix F, and C is the current source density. Since we use the same data V (i.e., the recorded data), the only important factor is the condition number of F. Since the condition number of F increases with increasing diameter because the data kernels become flatter, there is noise amplification for larger diameters. The iCSD method does not directly take into account the instability of the inverse problem. Instead, it mitigates noise by using a Gaussian spatial filter (see Pettersen et al., 2006), which is convolved with the estimated CSD from the unfiltered potentials and thus produces a spatially smoothed CSD estimate.

Our results show that changing the assumed diameter modifies the amplitudes of spontaneous current sources and sinks but that the spatiotemporal pattern remains similar (Figure 2; Table 1). Similarly, although changing the assumed diameter quantitatively modifies the computed PAC values, the laminar pattern of PAC is nevertheless qualitatively preserved for all assumed diameters of current sources and sinks (Figure 4 and Figures S3–S5).

## Conclusions

Our findings demonstrate both intra- and inter-laminar spontaneous PAC in a cortical column. Intra- and inter-laminar PAC involving layers 2/3–5a are higher than those involving layer 6. Our findings show that during spontaneous activity, delta, theta, and alpha oscillations are associated with periodic excitability. For the theta and alpha bands, this periodic excitability is lamina-dependent; on average, it is linked to dipoles of sinks and sources in the superficial layers and in layer 6, respectively, that are associated with increased excitability throughout the cortical column. Our findings emphasize differences between the function of layer 6 and those of superficial layers. Our study links recent theories on the involvement of PAC in resting-state functional connectivity with previous work showing lamina-specific thalamo-cortico-cortical anatomical connections.

## Author contributions

RS and AS initiated the study. AB developed the anesthesia regime. PK and MV developed the use of optical imaging of the probe's contacts. AB, SN, and VM acquired the data. RS, SN, and AS analyzed the data. RS, AB, and AS wrote the manuscript.

### Conflict of interest statement

The authors declare that the research was conducted in the absence of any commercial or financial relationships that could be construed as a potential conflict of interest.
