# Supplementary material for: Laminar Distribution of Phase-Amplitude Coupling of Spontaneous Current Sources and Sinks
Source: Front Neurosci. 2015 Dec 22;9:454. doi: 10.3389/fnins.2015.00454 (PMC4686797; doi:10.3389/fnins.2015.00454)
Supplement: Supplementary file 1 [file Presentation1.PDF]

Supplementary Material

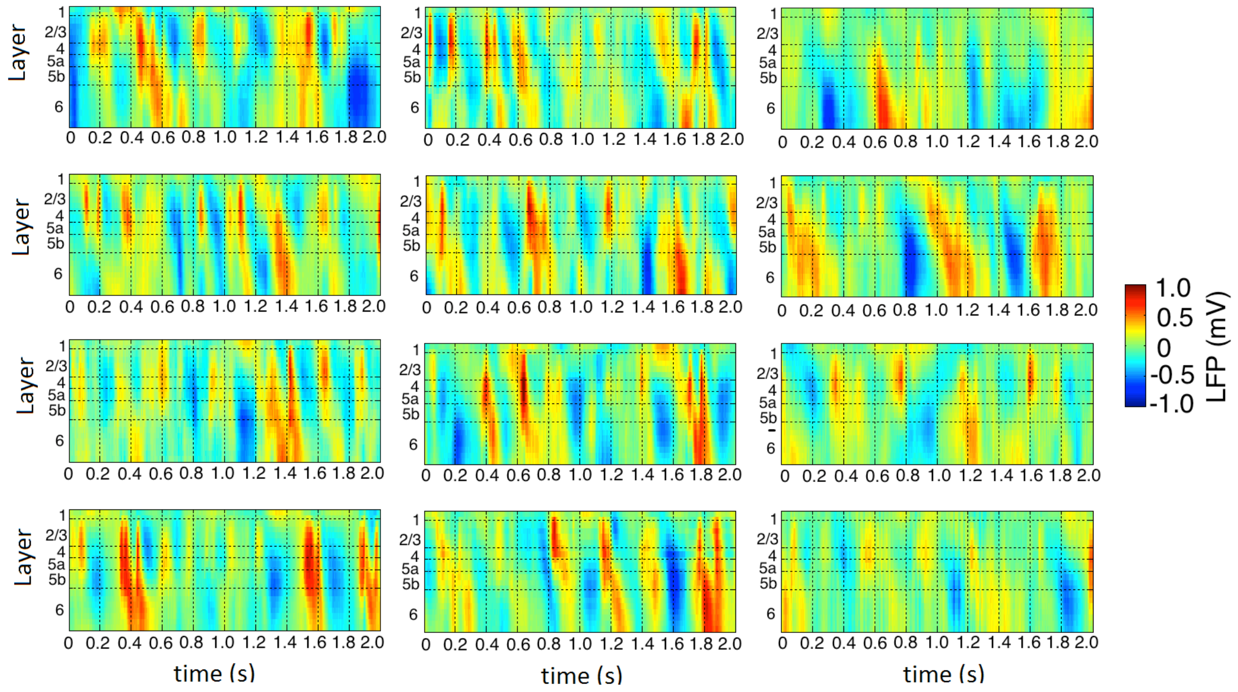

Fig. S1. Laminar distribution of local field potentials (LFP). Each panel shows 2 s LFP data from one of the 12 hemispheres used in this paper.

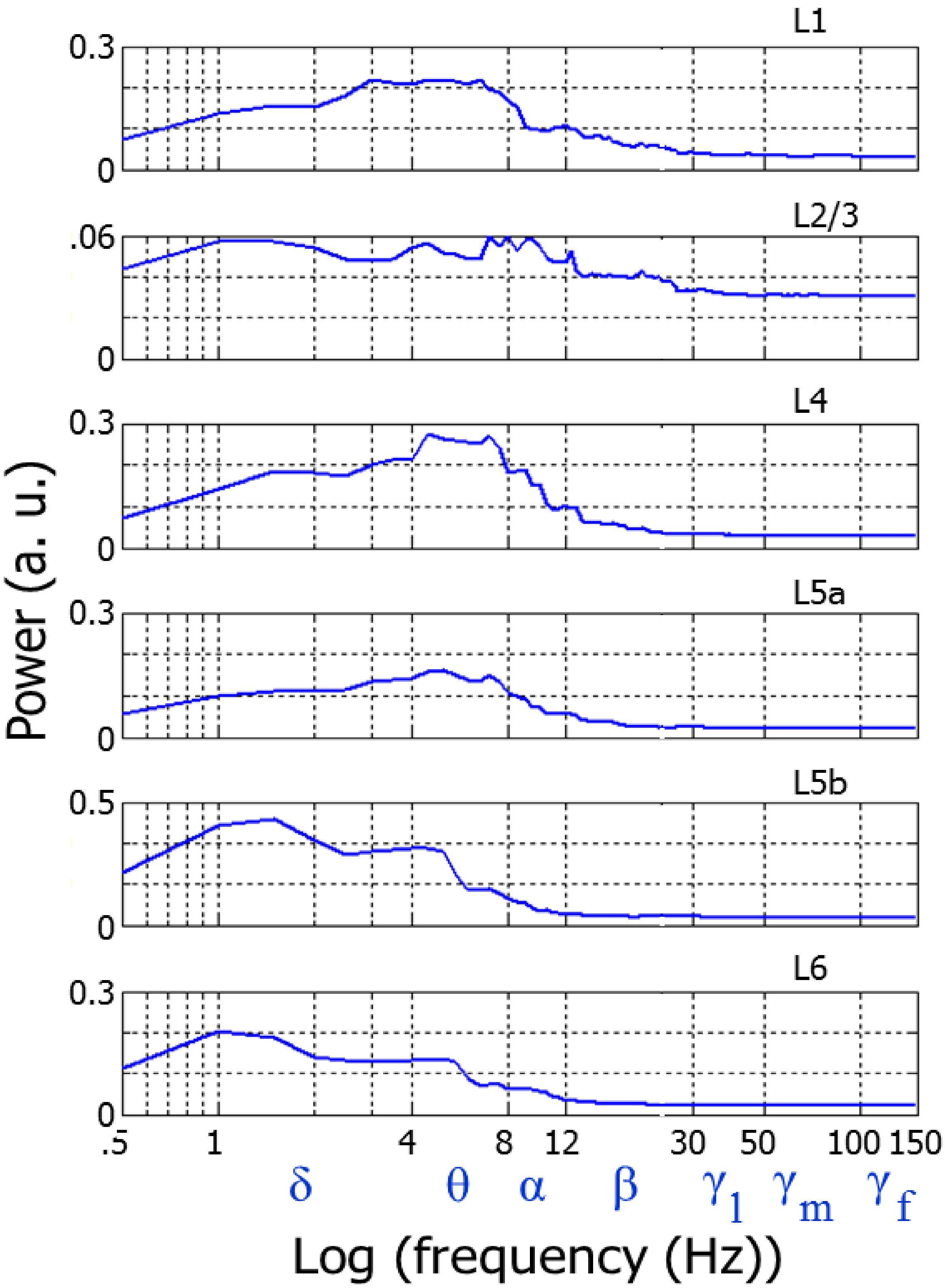

Fig. S2. Power spectrum of spontaneous CSD. The figure shows the average power spectrum across all 0.5 mm diameter iCSD 12 second segments, separately for each of the 6 cortical layers in one animal. Vertical dashed lines are used to separate bands from delta to fast-gamma.

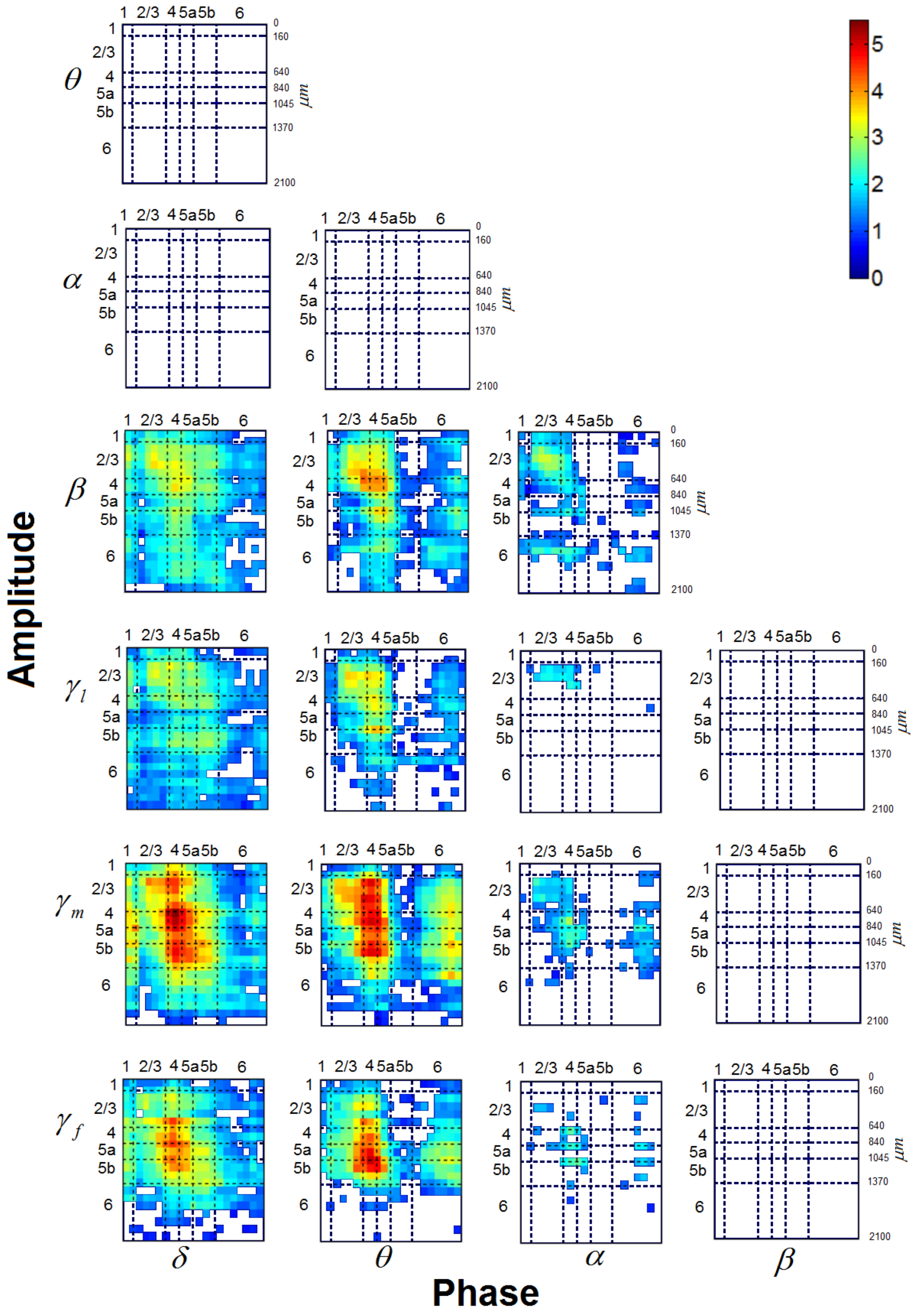

Fig. S3. Results of testing the null hypothesis that the average phase-amplitude coupling (PAC) is not different than zero. The figure shows the average Z score computed over 12 hemispheres (one Z-score per hemisphere) using iCSD and assumed 1.0 mm long diameters of current sources and sinks. Red: positive values. Blue: negative values. White entries stand for pairs of contacts that did not show statistical significant PAC.

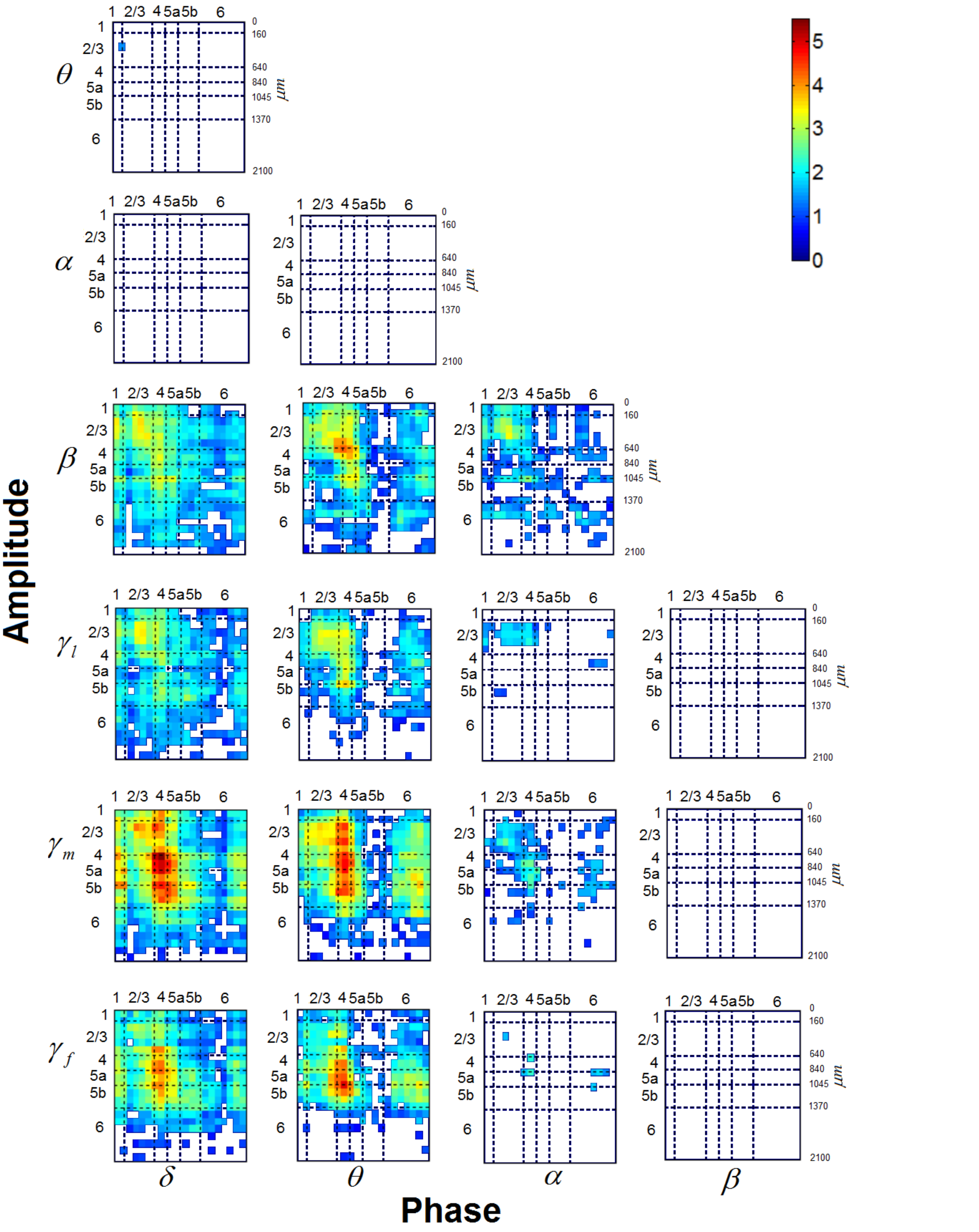

Fig. S4. Results of testing the null hypothesis that the average phase-amplitude coupling (PAC) is not different than zero. The figure shows the average Z score computed over 12 hemispheres (one Z-score per hemisphere) using iCSD and assumed 2.0 mm long diameters of current sources and sinks. Red: positive values. Blue: negative values. White entries stand for pairs of contacts that did not show statistical significant PAC.

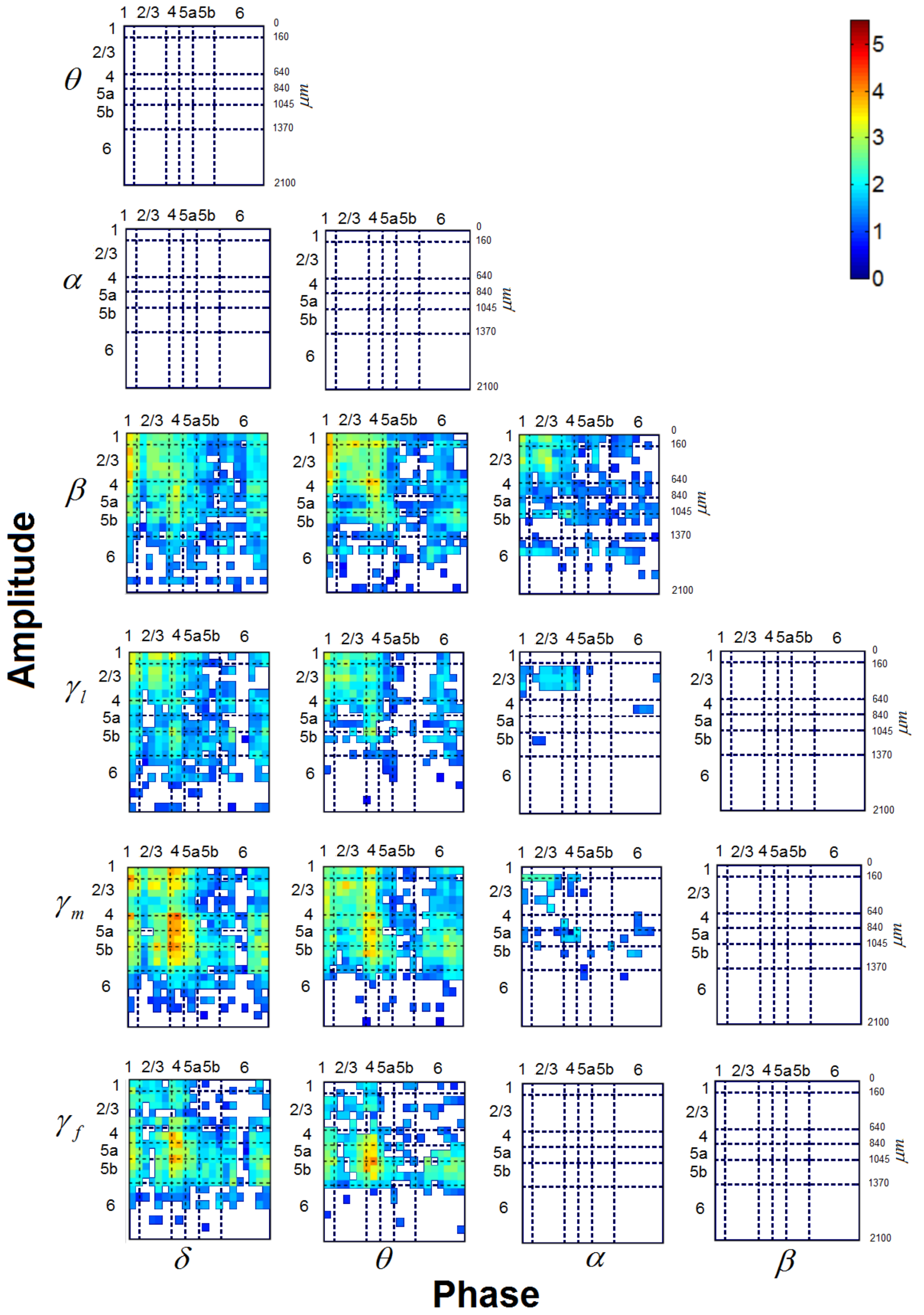

Fig. S5. Results of testing the null hypothesis that the average phase-amplitude coupling (PAC) is not different than zero. The figure shows the average Z score computed over 12 hemispheres (one Z-score per hemisphere) using standard CSD. Red: positive values. Blue: negative values. White entries stand for pairs of contacts that did not show statistical significant PAC.

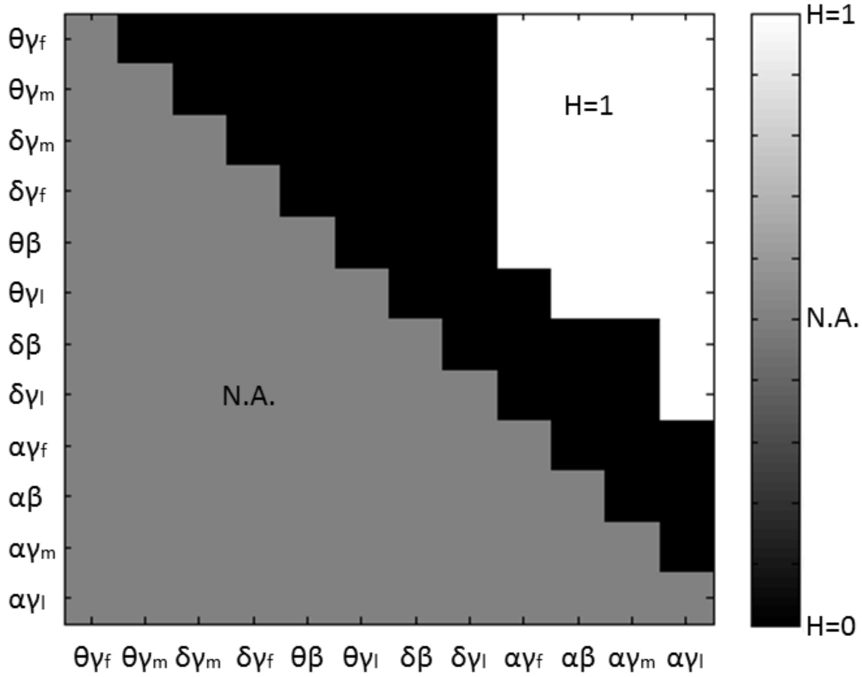

Fig. S6. Statistical testing for the ranking of statistically significant PAC combinations. No tests were performed for the entries of the diagonal and the left bottom part of the matrix (N.A., not applicable). Entries in dark show that the null hypothesis according to which the strength of the two compared connections is equal could not be rejected. For example, based on the data, we could not reject the hypothesis that the strength of $\theta\gamma_f$ is equal to that of $\theta\gamma_m$. White entries show that the PAC magnitude of the combination associated with the row was larger than that of the combination associated with the column. For example, the magnitude of PAC of $\theta\gamma_f$ was larger than those of $\alpha\gamma_f$, $\alpha\beta$, $\alpha\gamma_m$ and $\alpha\gamma_l$. Two tailed ttest, assuming that the variances of the two samples were unequal, and corrected for false discovery rate, $q \leq 0.05$.
